# Implication of GPRASP2 in the Proliferation and Hair Cell‐Forming of Cochlear Supporting Cells

**DOI:** 10.1111/cpr.13792

**Published:** 2024-12-15

**Authors:** Jing Cai, Kun Huang, Wenrui Li, Tianming Wang, Shen Yue, Zhibin Chen, Guangqian Xing, Qinjun Wei, Jun Yao, Xin Cao

**Affiliations:** ^1^ Department of Medical Genetics, School of Basic Medical Science Nanjing Medical University Nanjing China; ^2^ Central Laboratory, Translational Medicine Research Center The Affiliated Jiangning Hospital of Nanjing Medical University Nanjing China; ^3^ Department of Otolaryngology The First Affiliated Hospital With Nanjing Medical University Nanjing China; ^4^ Jiangsu Key Laboratory of Xenotransplantation Nanjing Medical University Nanjing China

**Keywords:** β‐catenin, GLI1, GPRASP2, hair cell, smoothened (SMO), supporting cell

## Abstract

G protein‐coupled receptor‐associated sorting protein 2 (*GPRASP2*) has been identified as the causative gene for X‐linked recessive syndromic hearing loss (SHL) in our previous study. However, the role of GPRASP2 in auditory function remains unclear. The present study demonstrated that *Gprasp2* overexpression in mouse organoids promoted the proliferation of supporting cells (SCs), which was mainly mediated by the Hedgehog signalling pathway. Meanwhile, GPRASP2 promoted hair cell (HC) formation from SCs via β‐catenin signalling. In addition, GPRASP2 deficiency resulted in increased lysosomal degradation of SMO protein, leading to decreased expression of β‐catenin and the Hedgehog pathway transcription factor GLI1. In neomycin‐treated mouse cochlear explant, the smoothened agonist (SAG) recured the HC loss and further facilitated AAV‐ie‐*Gprasp2* to promote the proliferation of SCs and formation of HCs. Our results suggested that GPRASP2 could be a potential candidate for gene therapy in the regeneration of HCs.

## Graphical abstract

Schematic diagram of GPRASP2‐mediated SCs proliferation and HCs formation.

1. GPRASP2 deficiency results in increased lysosomal degradation of SMO.
2. GPRASP2‐mediated SMO/GLI1 signalling promotes SC proliferation, which contributes to HC formation.
3. GPRASP2‐mediated SMO/β‐catenin signalling is implicated in HCs fate specification and differentiation.

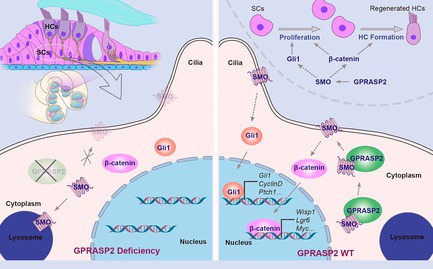

## 1 Introduction

Impairment or loss of cochlear hair cells (HCs) is one of the main causes of sensorineural hearing loss [1, 2]. Unfortunately, hearing loss caused by HC damage is commonly irreversible because mammalian inner ear HCs are not regenerative. However, recent studies have revealed that cochlear supporting cells (SCs) adjacent to HCs in perinatal mice have limited capacity to transdifferentiate into HCs [3, 4, 5]. Therefore, cochlear HC regeneration could be used to treat deafness caused by HC loss [6]. Although SCs provide a rapid and early source for the regeneration of HCs, their effect is limited because as each new HC is generated, one SC is lost [7, 8]. Therefore, the re‐entry of SCs into cell cycle proliferation is aimed at generating new HCs and replacing lost SCs owing to direct transdifferentiation. The gene regulation of HC regeneration is very similar to development; cochlear HCs and SCs originate from a common pool of progenitor cells termed prosensory cells [9, 10]. SRY‐box transcription factor 2 (SOX2) is essential for presensory cells, and the loss of SOX2 inhibits the formation of HCs and SCs [11, 12, 13]. However, during HC differentiation, the expression of SOX2 must be downregulated, and the expression of atonal BHLH transcription factor 1 (ATOH1) is activated [14, 15, 16]. Multiple signalling pathways are involved in regulating this process, including the Wnt, Notch1 and Hedgehog signalling pathways [17, 18]. In recent years, adeno‐associated virus (AAV) was used to mediate these regulatory factors and modulate the proliferation of SCs [19, 20].

In our previous study, the GPRASP2 mutation p.A573N was cosegregated with the clinical phenotype of syndromic hearing loss (SHL) in male patients [21]. GPRASP2 is a member of the GPRASP protein family, which displays a broad spectrum of interactions with G protein‐coupled receptors (GPCRs) to perform their biological functions, and the conserved 250 residues C‐ter domain of GASPs is composed of several armadillo‐like repeats [22, 23]. GPRASP2 interacts with Smoothened (SMO), the GPCR of the Sonic Hedgehog signalling pathway. GPRASP2 deficiency leads to a reduction in ciliary SMO, thus inhibiting the transduction of the Hedgehog signalling pathway [24]. The ligand Shh relieves the inhibitory effect of Patched 1 (PTCH1) on SMO, which enters primary cilia from the cell membrane. Subsequently, GLI transcriptional activators (GLI1, GLI2A and GLI3A) enter the nucleus to initiate the transcription of target genes [25]. Notably, disruption of GPRASP2 downregulates Hedgehog signalling and leads to apoptosis in auditory cells [26]. Persistent activation of the Hedgehog signalling pathway maintains a progenitor state of SOX2‐expressing prosensory cells and blocks HC formation [27, 28]. Because stimulating the proliferation of SCs or other prosensory cells is a pre‐requisite for the regeneration of HCs, the activation of the Hedgehog pathway provides a theoretical basis for HC regeneration. Furthermore, SMO overexpression has been reported to promote the proliferation of SCs and subsequent regeneration of HCs after neomycin‐mediated damage to the sensory epithelium in cochlear explants [29]. GPRASP2 participates in the regulation of Hedgehog pathway transduction; however, its role in the regeneration of HCs remains unknown.

In this study, we explored the function of GPRASP2 in cochlear SC proliferation and HC formation and its potential molecular mechanism using cochlear explants and organoids. GPRASP2 promoted the proliferation of SCs dependent on GLI1 and the formation of HCs dependent on β‐catenin, GPRASP2 deficiency leads to SMO degradation and down‐regulation of GLI1 and β‐catenin.

## 2 Materials and Methods

### 2.1 Animals and Cells Culture

All wild‐type (WT) C57BL/6 mice were purchased from the Production Department of the Experimental Animal Center at Nanjing Medical University (Nanjing, China). The house ear institute‐organ of Corti 1 (HEI‐OC1) cells that originated from male mice's Corti sensory cells were kindly provided by Dr. Federico Kalinec (The Regents of the University of California, USA). *Gprasp2*‐KO HEI‐OC1 cells were generated with CRISPR/Cas9 technology in our previously published study [26]. Briefly, two guide RNAs (gRNAs) were designed targeting the exon7 in *Gprasp2* and recombined into pX330 plasmid (AddGene). The sgRNA sequences are 5′‐GCAGGCCTAAAACCGATGCCA‐3′; 5′‐GCAAGGAGGTTTGAATTCGA‐3′. HEI‐OC1 cells were transfected with the Cas9‐sgRNA recombinant vectors and treated with G418, then the HEI‐OC1 cells that survived were seeded in 96‐well plates at 1 cell per well. Positive single‐cell colonies were selected by genotyping and Western blot (WB) assays. WT and *Gprasp2*‐KO HEI‐OC1 cells were cultured in Dulbecco's modified Eagle's medium (DMEM), supplemented with 10% FBS at 33°C and 10% CO_2_. HEK293Ta cells were cultured in DMEM supplemented with 10% FBS at 37°C and 5% CO_2_.

### 2.2 siRNA, Plasmids, and Reagents

pXJ40‐Flag, pXJ40‐HA, and m*Ctnnb1*‐MYC were purchased from the MiaoLing Plasmid Sharing Platform. Full‐length mouse *Gprasp2*, *Smo*, *Gli1* and *Tmed2* cDNA were generated by PCR with Flag or HA‐tagged to the N‐terminus and cloned into plasmids.

siRNAs specific for the mouse SMO were purchased from GenePharma (China). The siRNA sequences were listed in Table S1. Details of reagents used in the study are listed in Table S2.

### 2.3 Viruses

For *Gprasp2* knockdown experiments, short hairpin RNA (shRNA) sequence and negative control were synthesised and cloned into the GV112 vector (hU6‐MCS‐shRNA‐CMV‐Puromycin). Plasmids construction and lentivirus production were completed by GeneChem (China). *Gprasp2* (NM_001163015.2) was obtained from the cDNA library of OBiO Technology (China) and cloned into the lentivirus vector plasmid (pClenti‐EF1‐Neo‐CMV‐*Gprasp2*‐WPRE) or the AAV (serotype: AAV‐ie) vector plasmid (pcAAV‐CMV‐*Gprasp2*‐Flag‐WPRE). All plasmids construction, *Gprasp2* overexpressing lentiviruses and AAVs production were completed by OBiO Technology (China). The sequences of the shRNAs were as follows: Lenti‐sh*Gprasp2*‐1: 5′‐TCTGGGTTTCTCGCCTTATTA‐3′; Lenti‐sh*Gprasp2*‐2: 5′‐GACAAGGAAGAGCCTAATAAG‐3′; Lenti‐Vector (negative control): 5′‐TTCTCCGAACGTGTCACGT‐3′.

### 2.4 Cochlear Explants Culture

P3 mice were euthanized by CO_2_ asphyxiation and placed in 75% ethanol. Cochlear sensory epithelia were then isolated in chilled Hanks' balanced salt solution (HBSS) and cultured as cochlear explants in collagen‐coated 35 mm dishes in culture medium consisting of DMEM/F12 medium supplemented with GlutaMAX I, B27, N2 and 50 IU/mL penicillin G. After 12 h, fresh culture medium was replaced with CHIR99021 (3 μM), and culture medium was replaced every 2 days. For *Gprasp2* overexpression, cochlear explants were cultured in a medium with 2 × 10^10^ VG/mL AAVs for 48 h.

### 2.5 3D Cochlear Organoid Culture

The 3D cochlear organoid culture was established using a modified published protocol [30]. Briefly, cochlear sensory epithelium was isolated from P3 mice and then treated with 0.125% trypsin for 10 min at 37°C. The enzymatic reaction was terminated with DMEM/F12 medium containing 20 mg/mL trypsin inhibitor and 200 units/mL DNase I, and single cells were harvested by filtration through 40 μm filters. Cells were cultured in 96‐well ultra‐low attachment surface plates with growth medium consisting of DMEM/F12, 2% Matrigel, 0.02 mg/mL laminin, GlutaMAX I, N2, B27, 50 ng/mL murine EGF, 50 ng/mL murine FGF, 50 ng/mL murine IGF‐1, 3 μM CHIR99021, 1 mM VPA, 100 μg/mL pVc and 2 μM 616,452. On day 10 (D10), these organoids were switched to a differentiation medium consisting of DMEM/F12, GlutaMAX I, N2, B27, 3 μM CHIR99021, and 10 μM LY411575. During D0‐D10, organoids were split 1:2 every 2 days; during D10–D20, half of the medium was replaced every 2 days. For the cell proliferation assay of organoids, EdU (RiboBio, China) was added to the culture medium at a concentration of 10 μM 24 h before sample collection. For *Gprasp2* overexpression or knockdown, Lenti‐*Gprasp2* or Lenti‐sh*Gprasp2* was added to the cochlear organoid medium at a concentration of 2 × 10^6^ TU/mL on D0.

### 2.6 Immunohistochemical Staining

Cochlear tissue from P1 and P7 WT C57BL/6 mice were analysed for GPRASP2 by immunohistochemistry, following standard procedures using a rabbit two‐step detection kit (ZSGB‐BIO). The primary antibody is rabbit anti‐GPRASP2 (1:1000, ab253010, Abcam).

### 2.7 Immunoblotting and Co‐Immunoprecipitation

Cells or cochlear tissues were lysed in RIPA buffer with protease and phosphatase inhibitors (MCE, China) for 1 h at 4°C. The lysate was ultrasonicated with an ultrasonic cell breaker, and centrifuged for 20 min at 14,000 × *g*. The protein concentrations of supernatants were qualified by BCA protein quantification assay (Beyotime, China). The samples were supplemented with 5× SDS loading buffer, incubated for 5 min at 95°C, separated by SDS‐PAGE, and followed by WB analysis. In addition, the Membrane and Cytoplasmic Protein Extraction Kit (Beyotime, China) was used to separate the cell membrane protein and cytoplasmic protein of HEI‐OC1 cells. Co‐immunoprecipitation (CO‐IP) was performed using the Immunoprecipitation Kit (Beyotime, China). The following antibodies were used: rabbit GAPDH (1:2000, 5174S; CST); rabbit GPRASP2 (1:1000, ab253010; Abcam); rabbit GLI1 (1:1000, 2534; CST); goat GLI3(1:1000, AF3690; RD); mouse SMO (1:500, sc‐400491; Santa Cruz); rabbit β‐catenin (1:1000, 51067‐2‐AP; Proteintech); rabbit HA (1:1000, 3724S; CST); rabbit Flag (1:1000, 14793S; CST); mouse actin (1:2000, 66009‐1; Proteintech); rabbit Ezrin (1:1000, ab133297; Abcam); HRP‐conjugated Anti‐Mouse IgG (1:5000, SA00001‐1; Proteintech); HRP‐conjugated Anti‐Rabbit IgG (1:5000, SA00001‐2; Proteintech).

### 2.8 RNA Extraction and Quantitative Real‐Time PCR

Total RNAs from cells and cochlear tissues were extracted using the SteadyPure RNA Extraction Kit (AGbio, China) and reverse‐transcribed into cDNA using the All‐in‐One First‐Strand cDNA Synthesis SuperMix for qPCR Kit (TransGen, China). Real‐Time PCR (RT‐PCR) was carried out in triplicate using the Green qPCR SuperMix Kit (TransGen, China), and relative gene expression was calculated using the ΔΔ*C*_t_ method. The qPCR primer sequences are listed in Table S1.

### 2.9 Immunofluorescence Staining and Confocal Imaging

HEI‐OC1 cells, cochlear organoids, explants, or basilar membranes dissected from neonatal mice were fixed with 4% paraformaldehyde (PFA) in PBS for 20 min at room temperature. Permeabilization and blocking were performed with 0.1% Triton X‐100 with 10% normal goat serum for 1 h at room temperature. The samples were then incubated with the primary antibodies overnight at 4°C and incubated with secondary antibodies conjugated with Alexa Fluor for 2 h at room temperature. Subsequently, cell nuclei were visualised with DAPI. Fluorescence images were visualised on the Zeiss Lsm880 confocal microscope and analysed using ZEN2 software. The following antibodies were used: rabbit GPRASP2 (1:200, ab253010; Abcam); mouse SMO (1:50, sc‐400491; Santa Cruz); rabbit HA (1:200, 3724S; CST); mouse MYO7A (1:100, sc‐74516; Santa); SOX2 (1:100, sc‐365832; Santa); rabbit CTBP2 (1:100, ab128871; Abcam); WGA‐488 (1:200, W11261; Invitrogen); DAPI (AB104139; Abcam); rabbit UB (1:500, ET1609; Huabio); P62 (1:1000, A19700; Abclonal); Alexa Fluor 488 anti‐Mouse IgG (1:200, A‐21202; Invitrogen); Alexa Fluor 555 anti‐Mouse IgG (1:200, A‐21422; Invitrogen); Alexa Fluor 488 anti‐Rabbit IgG (1:200, A‐31565; Invitrogen); Alexa Fluor 594 anti‐Rabbit IgG (1:200, 8889S; CST); Alexa Fluor 594 anti‐Mouse IgG2a (1:200, A21135; Invitrogen); (1:200, A21121; Invitrogen); Alexa Fluor 647 anti‐Mouse IgG1 (1:200, A21240; Invitrogen); Alexa Fluor 488 anti‐Mouse IgG1 (1:200, A‐21121; Invitrogen).

### 2.10 Flow Cytometric Analysis for Cell Cycle

HEI‐OC1 cells were collected and fixed with cooled 75% ethanol, and cell cycle distribution was analysed by the flow cytometer (BD Biosciences, USA). Cochlear organoid cells were harvested by organoid digestion solution (Absin, China) and fluorescently stained with anti‐SOX2 and fluorescent secondary antibody, and then SOX2^+^ cells sorted by flow cytometry were subjected to cell cycle analysis.

### 2.11 Count Kit‐8 Assays

Treated HEI‐OC1 cells were seeded in 96‐well plates at 4000 cells/well density. Cell proliferation was then determined using the CCK‐8 kit (MCE, China) at different time points. The following formula was used for calculations:

$$\text{Relative cell proliferation}=\frac{\mathrm{OD}\;\left(\text{experiment}\right)–\mathrm{OD}\;\left(\text{blank}\right)}{\mathrm{OD}\;\left(\text{control}\right)–\mathrm{OD}\;\left(\text{blank}\right)}$$

### 2.12 EdU Incorporation Assays

HEI‐OC1s were seeded on 14 mm coverslips, cultured in medium overnight, and incubated with 50 μM EdU for 2 h to label proliferative cells. Cochlear organoids and explants were incubated with 10 μM EdU for 24 h and 6 days. Then, fluorescence staining of proliferating cells was performed using the EdU Cell Proliferation Assay Kit (RiboBio, China). Images were taken with a fluorescence microscope, and the percentage of EdU‐positive cells was measured in five different fields.

### 2.13 RNA Sequencing

RNA sequencing data of *Gprasp2*‐KO and WT HEI‐OC1 cells from our previous study [26]. The clusterProfiler R package was used for Kyoto Encyclopedia of Genes and Genomes (KEGG) pathway enrichment analysis.

### 2.14 Statistical Analysis

Statistical analysis was performed using GraphPad Prism 8.0. One‐way ANOVA was used to compare between indicated groups, and *p* < 0.05 were considered significant. Each measurement was repeated at least three times to avoid bias and is presented as mean ± standard error (SEM).

## 3 Results

### 3.1 GPRASP2 Promotes SC Proliferation in Cochlear Organoids

**FIGURE 1:**
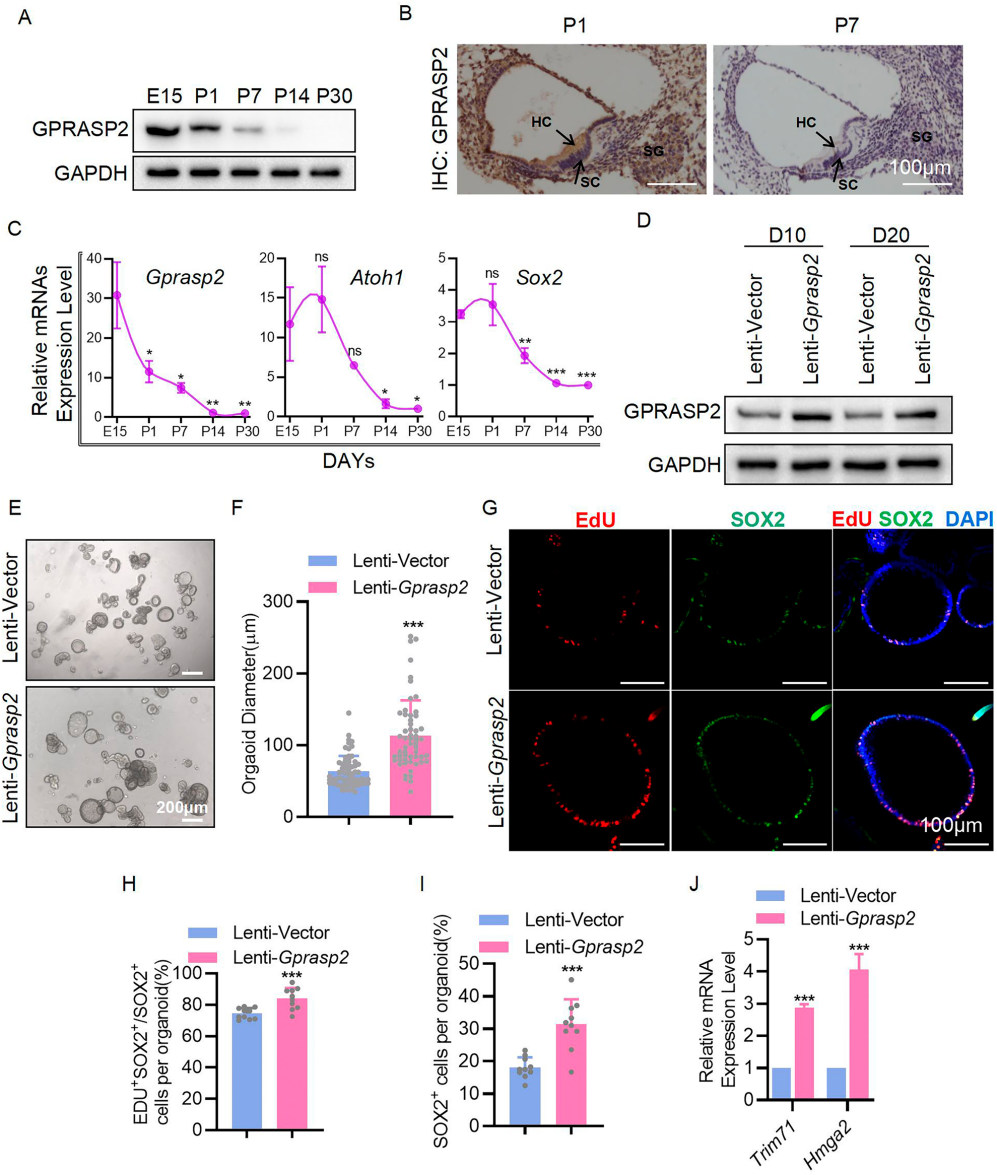
GPRASP2 promotes SC proliferation in cochlea organoids. (A) WB detection of GPRASP2 protein levels in the mouse cochlea at E15, P1, P7, P14, P30 and GAPDH were used as internal controls. (B) IHC staining of GPRASP2 in the cochlea of WT P1 and P7 mice. (C) mRNA levels of *Gprasp2*, *Atoh1* and *Sox2* in the mouse cochlea at E15, P1, P7, P14 and P30. *n* = 3, each sample contains four cochleae. (D) WB assay of GPRASP2 expression in mouse cochlear organoids infected with Lenti‐*Gprasp2*, and the Lenti‐Vector‐infected group was used as control. (E) Representative images of cochlear organoids transfected with Lenti‐Vector or Lenti‐*Gprasp2* at D10. Scale bar = 200 μm. (F) Organoid diameter in (E). *n* ≥ 50. (G) Immunofluorescence staining of cochlear organoids with EdU (red), SOX2 (green), and DAPI (blue) at D10 after transduction with Lenti‐Vector or Lenti‐*Gprasp2*. Scale bar = 100 μm. (H, I) Percentage quantification of EdU^+^SOX2^+^ cells and SOX2^+^ cells per organoid in (G). SOX2^+^EDU^+^/SOX2^+^ cells per cochlear organoid (%) = (SOX2^+^EDU^+^ cells)/(SOX2^+^ cells) × 100. SOX2^+^ cells per cochlear organoid (%) = (SOX2^+^EDU^+^ cells)/(DAPI^+^ cells) × 100. *n* ≥ 10. (J) RT‐PCR detection of *Trim71* and *Hmga2* mRNAs in cochlear organoids transfected with Lenti‐Vector or Lenti‐*Gprasp2*. *n* = 3, each sample contains roughly 150 organoids. Data in bar graphs are presented as mean ± SEM. *p < 0.05, **p < 0.01, ***p < 0.001, ns. not significant.

To investigate the physiological role of GPRASP2 in the auditory system, its expression was examined at various stages of cochlear development, from the embryonic (E) to postnatal (P) periods (E15, P1, P7, P14 and P30) (Figure 1A). GPRASP2 expression significantly decreased over time in the postnatal period and was nearly absent on Day 7 (Figure 1A,B). The *Gprasp2* mRNA expression trend aligned with that of *Atoh1* and *Sox2*, exhibiting a notable postnatal decrease (Figure 1C). GPRASP2 was expressed at high levels during the embryonic and early postnatal periods, indicating its pivotal role in these phases. Therefore, we speculated that GPRASP2 plays an important role in the proliferation and maturation of cells during cochlear development.

To further investigate the regulatory effect of GPRASP2 on cochlear SC and HC fates, we cultured cochlear organoids for 10 days (D0–D10) for proliferation and 10 days (D10–D20) for induced differentiation (Figure S1A). The mRNA expression of HC markers, including *Atoh1*, Myosin VIIA (*Myo7a*) and POU Class 4 Homeobox 3 (*Pou4f3*), was significantly increased in D20 organoids. In contrast, the expression of *Sox2* and Lunatic Fringe (*Lfng*), markers of cochlear SCs, was effectively reduced (Figure S1B). It has been suggested that cochlear organoids can successfully express HC marker genes by induction. *Gprasp2* overexpression (by lentivirus) was induced on D0 in the organoid culture (Figure 1D). The average diameter of organoids at D10 was larger than that of the control group (Figure 1E,F), and the number of proliferating SCs (EdU^+^SOX2^+^) was significantly increased in the *Gprasp2* overexpression group based on EdU assay results (Figure 1G,H) and the total number of SOX2^+^ cells was markedly higher than that in the control group (Figure 1I). In addition, the expression of stemness markers *Trim71* and *Hmga2* significantly increased (Figure 1J). Consistent with these results, Lenti‐sh*Gprasp2* (Figure S1C) reduced the diameter of D10 organoids (Figure S1D,E), and the expression of *Trim71* and *Hmga2* (Figure S1F). These results indicate that GPRASP2 can promote the proliferation of SCs in cochlear organoids. To further verify this phenomenon, *Gprasp2* was overexpressed in P3 mouse cochlear explants using AAV‐ie‐*Gprasp2* (Figure S2A,B). Immunofluorescence analysis revealed that in the cochlear explants of the AAV‐ie‐*Gprasp2* group, the number of SOX2‐labelled SCs in the apical turn was significantly higher than in the control group, while the increase in SCs in the middle turn was minimal. No difference was observed in the number of SCs in the basal turn between the AAV‐ie‐*Gprasp2* and control groups. In addition, the number of MYO7A‐labelled HCs showed the same trend (Figure S2C–E). Taken together, these data indicate that *Gprasp2* overexpression promotes the proliferation of SCs and the subsequent formation of HCs in the sensory area of the apical‐middle turn of cochlear explants and enhances SC proliferation in cochlear organoids.

### 3.2 GPRASP2 Promotes HC Formation From SCs in Cochlear Organoids

**FIGURE 2:**
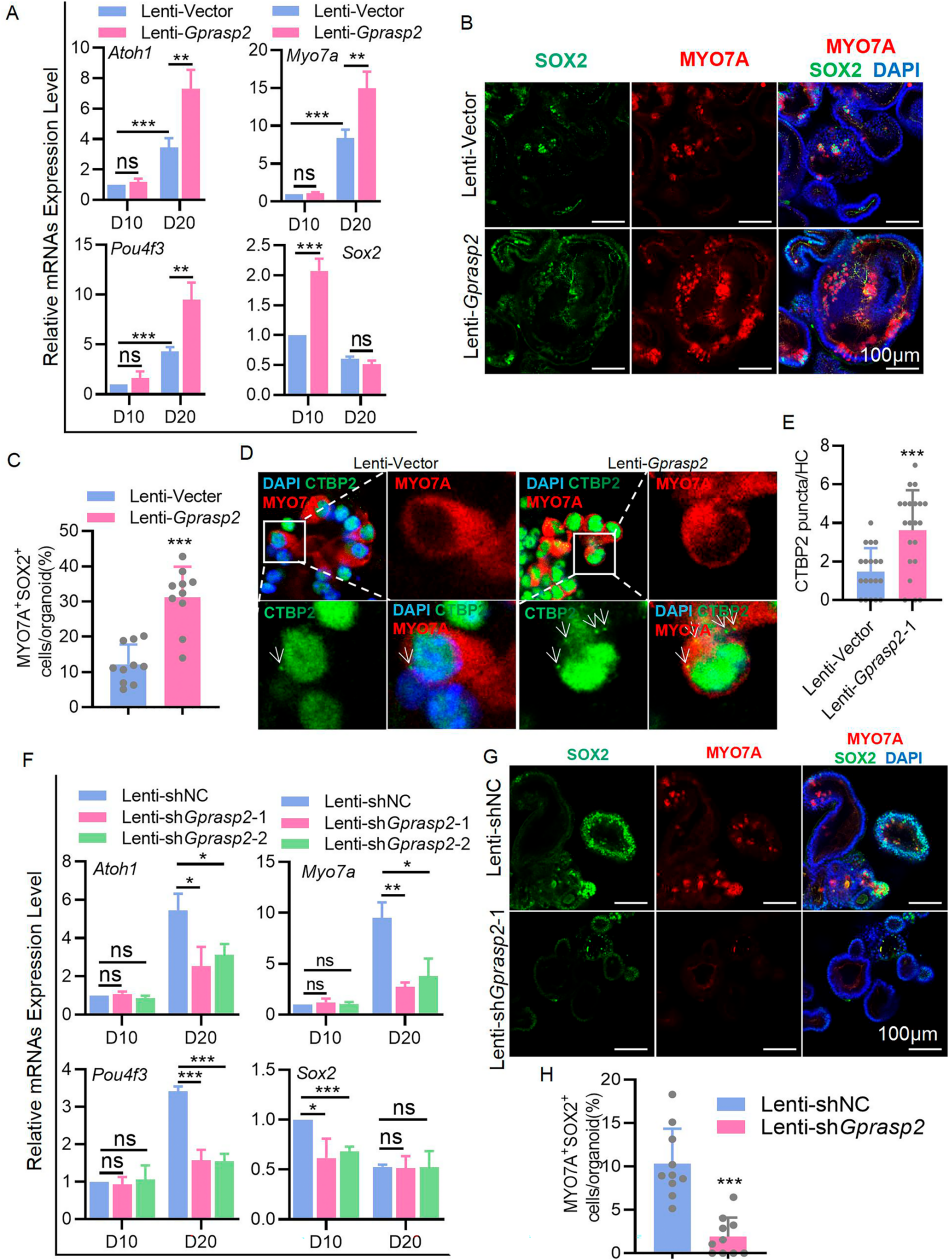
GPRASP2 promotes HC formation in cochlear organoids. (A) RT‐PCR detection of *Atoh1*, *Myo7a*, *Pou4f3* and *Sox2* mRNAs in cochlear organoids transfected with Lenti‐Vector or Lenti‐*Gprasp2*. *n* = 3, each sample was generated by approximately 150 organoids. (B) Immunofluorescence staining of cochlear organoids at D20 with MYO7A (red), SOX2 (green) and DAPI (blue) after transfection with Lenti‐Vector or Lenti‐*Gprasp2*. Scale bar = 100 μm. (C) Percentage quantification of MYO7A^+^SOX2^+^ cells per organoid in (B). *n* ≥ 10. (D) Immunofluorescence staining of cochlear organoids at D20 with MYO7A (red), CTBP2 (green) and DAPI (blue) after transfection with Lenti‐Vector or Lenti‐*Gprasp2*. (E) Comparison of CTBP2 ribbon puncta number of MYO7A^+^ cells in Lenti‐*Gprasp2* organoids with the control group in (D). *n* ≥ 15. (F) RT‐PCR detection of *Atoh1*, *Myo7a*, *Pou4f3* and *Sox2* mRNAs in cochlear organoids transfected with Lenti‐shNC, Lenti‐sh*Gprasp2*‐1 or Lenti‐sh*Gprasp2*‐2. *n* = 3, each sample was generated by approximately 150 organoids. (G) Immunofluorescence staining of cochlear organoids at D20 with MYO7A (red), SOX2 (green) and DAPI (blue) after transfected with Lenti‐shNC or Lenti‐sh*Gprasp2*‐1. Scale bar = 100 μm. (H) Percentage quantification of MYO7A^+^SOX2^+^ cells per organoid in (G), *n* ≥ 10. Data in bar graphs are presented as mean ± SEM. *p < 0.05, **p < 0.01, ***p < 0.001, ns. not significant.

To validate the effect of GPRASP2 on HC formation, *Gprasp2* was overexpressed in D0 cochlea organoids and induced to differentiate during D10 to D20. The expression of the HC markers *Atoh1*, *Myo7a* and *Pou4f3* increased (Figure 2A). Mature cochlear HCs are known to be SOX2 negative and MYO7A positive, and the detection of MYO7A and SOX2 double‐positive cells (MYO7A^+^SOX2^+^) in cochleae indicated the newly formed HCs from SCs [31, 32]. Immunofluorescence results showed that the number of newly formed HCs (MYO7A^+^SOX2^+^) was significantly higher in Lenti‐*Gprasp2* organoids than in the control group (Figure 2B,C). *Sox2* expression in the *Gprasp2*‐overexpression group (Lenti‐*Gprasp2*) was higher than that in the control group (Lenti‐Vector) at D10, but no significant difference was observed between the *Sox2* expression of the two groups at D20 (Figure 2A). In the *Gprasp2* overexpression group, the level of *Sox2* mRNA decreased by 75% (2.07–0.51) after the induction of differentiation, while the control group decreased by only 39% (1–0.60), indicating that more SCs in the overexpressed *Gprasp2* cochlear organoids were induced to differentiate into HCs (Figure 2A). In addition, the overexpression group exhibited significantly more CTBP2 punctate signals (representing active synapses) in MYO7A^+^ cells than in the control group (Figure 2D,E). Similar results were observed after *Gprasp2* knockdown, which decreased the expression of *Atoh1*, *Myo7a* and *Pou4f3* (Figure 2F) and decreased the number of MYO7A^+^SOX2^+^ cells (Figure 2G,H). Furthermore, reduced *Sox2* expression was noted in the organoids of the *Gprasp2*‐knockdown group at D10, with a milder reduction (16%: 0.61–0.51; 23%: 0.68–0.52) compared to that in the control group (47%: 1–0.52) after the induction of differentiation (Figure 2F). These results indicated that GPRASP2 effectively promoted the formation of HCs from SCs in cochlear organoids.

### 3.3 GPRASP2 Regulated SMO‐GLI1 Signalling Promotes SC Proliferation but Not HC Formation

**FIGURE 3:**
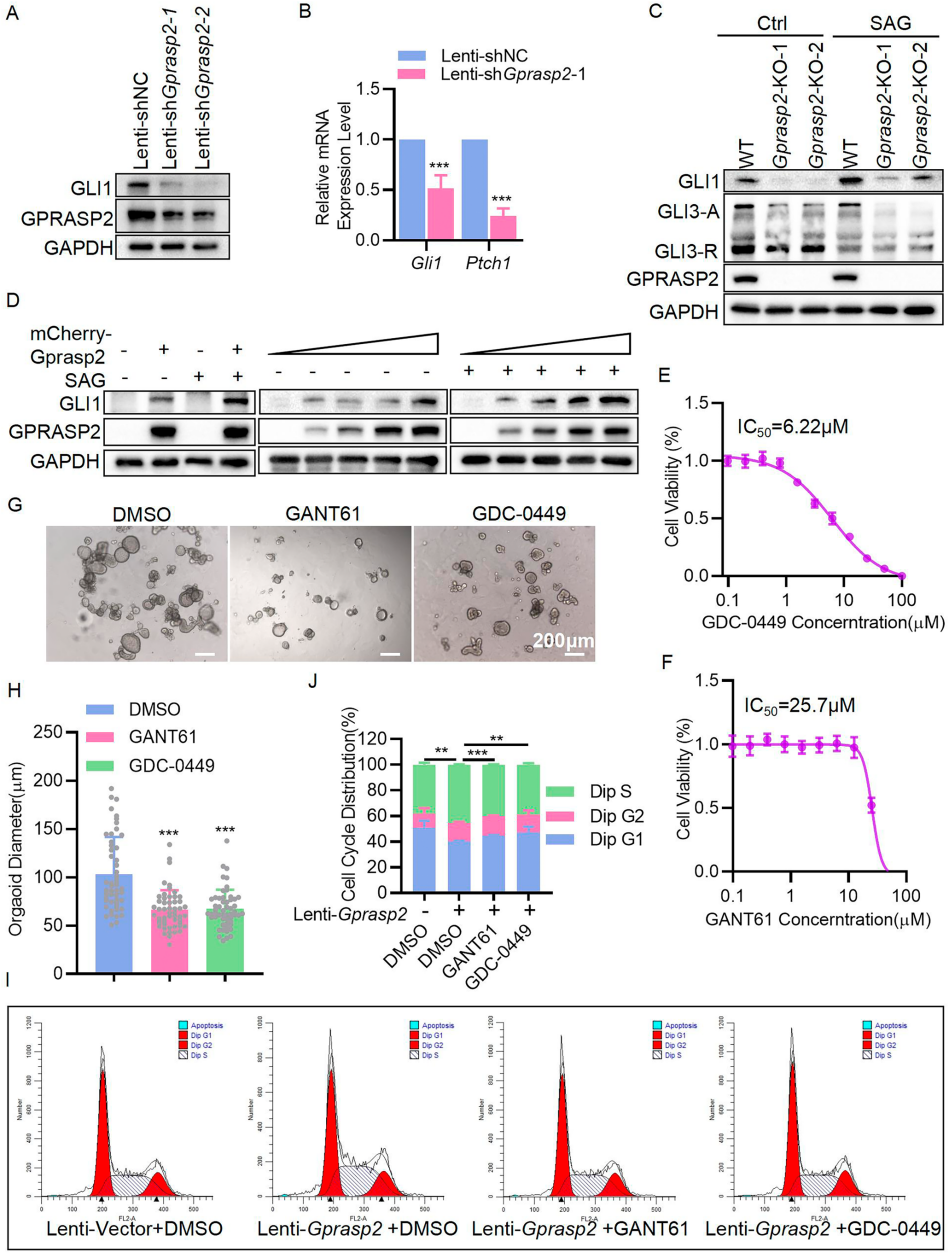
GPRASP2 regulated SMO‐GLI1 signalling promotes SC proliferation but not HC formation. (A) WB detection of GPRASP2 and GLI1 protein levels in cochlear organoids transfected with Lenti‐shNC, Lenti‐sh*Gprasp2*‐1 or Lenti‐sh*Gprasp2*‐2. Each sample was generated by approximately 150 organoids. (B) RT‐PCR detection of *Gli1* and *Ptch1* mRNAs in cochlear organoids transfected with Lenti‐shNC, Lenti‐sh*Gprasp2*‐1. *n* = 3, each sample was generated by approximately 150 organoids. (C) WB detection of GPRASP2, GLI1 and GLI3‐A/R protein levels in WT and *Gprasp2*‐KO HEI‐OC1 cells treated with SAG (200 nM) for 24 h. (D) WB detection of GPRASP2 and GLI1 protein levels in *Gprasp2*‐KO HEI‐OC1 cells treated with exogenous *Gprasp2* and SAG alone or together. (E, F) The relationship between cell viability and dose of GDC‐0449 and GANT61 was shown by the dose‐effect curve. (G) Representative images of D10 cochlear organoids treated with DMSO (1:1000), GANT61 (25 μM) or GDC‐0449(6 μM) from D1 to D10. Scale bar = 200 mm. (H) Organoid diameter in (G) *n* ≥ 50. (I) Flow cytometry detected the cell cycle of SOX2^+^ cell from D10 cochlear organoids transfected with Lenti‐Vector or Lenti‐*Gprasp2*, and the medium was supplemented with DMSO, GANT61 (25 μM), or GDC‐0449 (6 μM). (J) Quantification of three independent experiments as in (I). Data in bar graphs are presented as mean ± SEM. *p < 0.05, **p < 0.01, ***p < 0.001.

Previous reports have shown that GPRASP2 activates the Hedgehog signalling pathway by regulating SMO and that the Hedgehog pathway is essential for the proliferation of cochlear SCs and other stem cells [28, 33]. To verify that GPRASP2 regulates the Hedgehog pathway in cochlear organoids, we detected the transcription factor GLI1 in this pathway. The results showed that expression of the GLI1 protein was inhibited by *Gprasp2* knockdown (Figure 3A), and *Gli1* and *Ptch1* mRNA expression levels were significantly reduced (Figure 3B). To facilitate further study of its regulatory mechanisms, a cell line should be used for subsequent research. HEI‐OC1 is a cell line commonly used in auditory system research, and some studies have suggested that it could serve as a progenitor for sensory and SCs [26, 34]. We determined whether the regulation of GPRASP2 on the proliferation of HEI‐OC1 cells was consistent with that of SCs. We found that *Gprasp2* knockout in HEI‐OC1 cells (*Gprasp2*‐KO HEI‐OC1) using CRISPR–Cas9 technology resulted in a reduction in cell sphere‐forming ability (Figure S3A,B), proportion of cells in the S phase (Figure S3C,D), and the rate of EdU‐positive cells (Figure S3E,F). These results indicated that GPRASP2 deficiency can inhibit the proliferation of HEI‐OC1 cells, suggesting that HEI‐OC1 was a suitable cell model for this study. Consistent with the above results, activation of the Hedgehog pathway was also inhibited in *Gprasp2*‐KO HEI‐OC1 cells (Figure 3C), and GLI1 expression was rescued after restoring *Gprasp2* expression in *Gprasp2*‐KO HEI‐OC1 cells in a dose‐dependent manner (Figure 3D). These results further prove that GPRASP2 can regulate Hedgehog signalling in the cochlea, but whether this is the potential mechanism by which GPRASP2 regulates the proliferation of cochlear SCs remains uncertain.

To validate this hypothesis, we first used HEI‐OC1 cells to determine the inhibitory concentration 50 (IC_50_) of Hedgehog signalling pathway inhibitors GDC‐0449 (targeting SMO protein, IC_50_: 6 μM) and GANT61 (targeting GLI1 protein, IC_50_: 25 μM; Figure 3E,F). The results showed that both GDC‐0449 (6 μM) and GANT61 (25 μM) suppressed cochlear organoid growth (Figure 3G,H). Cell cycle analysis of SOX2^+^ cells sorted by flow cytometry from cochlear organoids at D10 showed that GPRASP2 promoted the proliferation of SCs, which was counteracted by GDC‐0449 (6 μM) and GANT61 (25 μM; Figure 3I,J). These results further confirmed that GPRASP2 promoted the proliferation of SCs via Hedgehog. Based on these data, we investigated whether GPRASP2/Hedgehog signalling can regulate HC formation. Then GANT61 (10 μM) and GDC‐0449 (1 μM) were introduced at the maximum concentration and did not affect proliferation at D10, and cochlear organoids were induced to differentiate for 10 days. GDC‐0449 significantly suppressed the expression of *Atoh1*, *Myo7a* and *Pou4f3*; however, the inhibitory effect of GANT61 was insignificant (Figure S4A). This result shows that the inhibition of SMO can inhibit HC‐related gene expression, but the inhibition of GLI1 does not, suggesting that the inhibition of SMO leads to the inhibition of HC formation not through GLI1 but through other downstream molecules. Although *Gprasp2* overexpression in organoids promoted GLI1 expression, it was significantly downregulated during the differentiation stage (Figure S4B). Furthermore, GLI1 expression was significantly decreased in the cochlea of mice at P1 compared to that during the embryonic stage and was barely detectable at P7 (Figure S4C). These results indicate that GPRASP2 promotes SCs through SMO‐GLI1 signalling, but GPRASP2 promotes HC differentiation via other mechanisms.

### 3.4 GPRASP2 Promotes HC Formation Dependent on β‐Catenin

**FIGURE 4:**
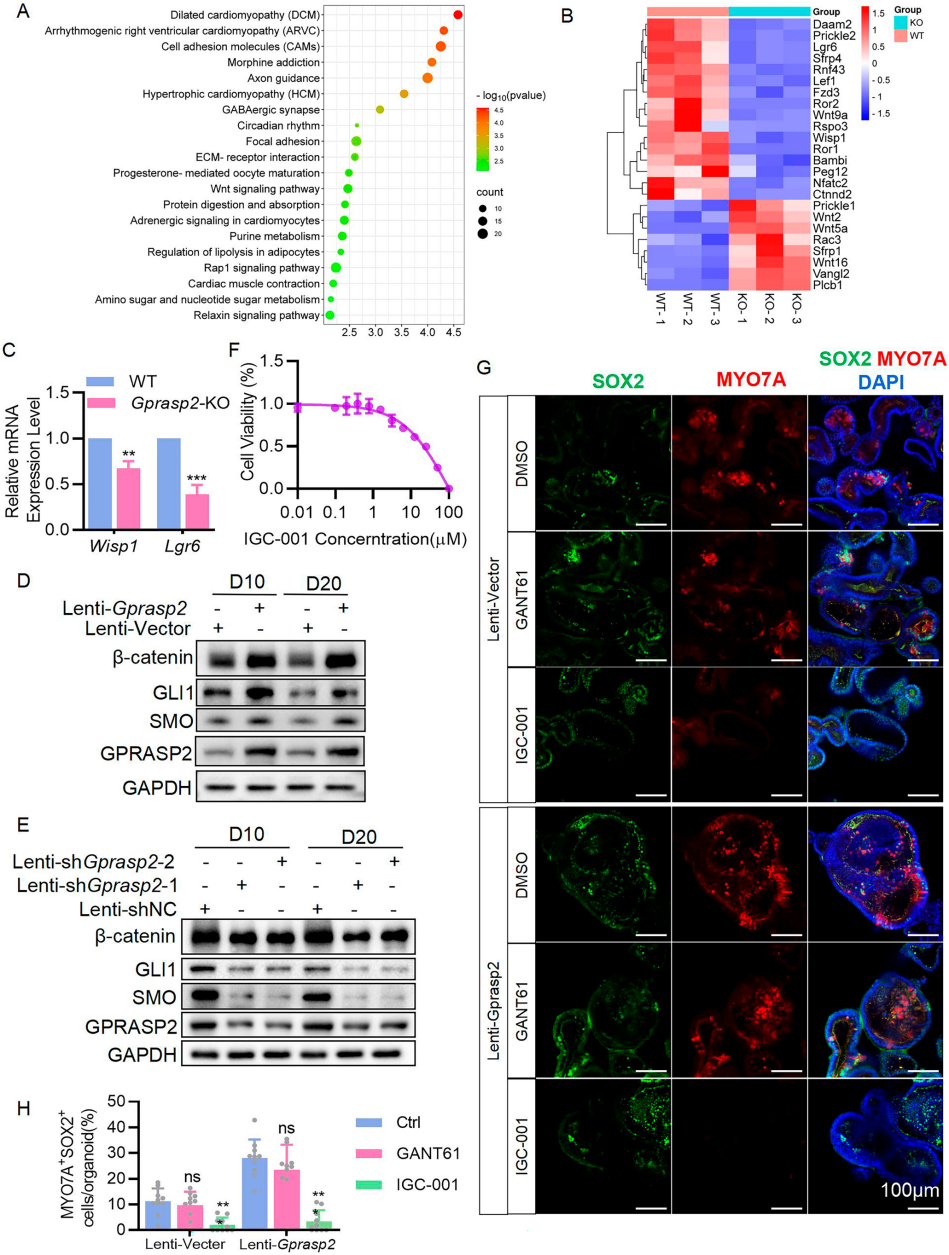
GPRASP2 promotes HC formation dependent on β‐catenin. (A) KEGG pathway enrichment of downregulated differentially expressed genes (DEGs) in *Gprasp2*‐KO HEI‐OC1. (B) Heatmap of genes in Wnt signalling pathway. (C) RT‐PCR detection of *Wisp1* and *Lgr6* mRNAs in WT and *Gprasp2*‐KO HEI‐OC1. *n* = 3. (D, E) WB detection of GPRASP2, GLI1, SMO and β‐catenin protein levels in cochlear organoids transfected with Lenti‐*Gprasp2* or Lenti‐Sh*Gprasp2* at D10 and D20. Each sample was generated by approximately 150 organoids. (F) The relationship between HEI‐OC1 cells viability and dose of IGC‐001 was shown by the dose‐effect curve. (G) Immunofluorescence staining of cochlear organoids with MYO7A (red), SOX2 (green) and DAPI (blue) after transfection with Lenti‐*Gprasp2* at D20 and treated with GANT61 or IGC‐001 starting at D10. Scale bar = 100 μm. (H) Percentage quantification of MYO7A^+^SOX2^+^ cells per organoid in (G). *n* ≥ 10. Data in bar graphs are presented as mean ± SEM. *p < 0.05, **p < 0.01, ns. not significant.

To unravel the potential mechanism by which GPRASP2 promotes HC formation, we conducted RNA‐seq analyses of WT and *Gprasp2*‐KO HEI‐OC1 cells. KEGG enrichment analysis of the genes for which expression was downregulated in *Gprasp2*‐KO HEI‐OC1 cells and observed the Wnt signalling pathway (Figure 4A). The expression levels of most of the differentially expressed genes associated with the Wnt pathway were downregulated in *Gprasp2*‐KO HEI‐OC1 cells, including leucine‐rich repeat‐containing G protein‐coupled receptor 6 (*Lgr6*), which is highly correlated with cochlear stem cells (Figure 4B). The mRNA levels of the β‐catenin target genes *Lgr6* and WNT1 inducible signalling pathway protein 1 (*Wisp1*) were significantly reduced in *Gprasp2*‐KO HEI‐OC1 cells (Figure 4C), while they were higher in organoids overexpressing *Gprasp2* than in the control group (Figure S4D,E). However, *Gli1* and *Ptch1* expression was significantly reduced during the differentiation stage (Figure S4F,G). These results indicate that GPRASP2 is involved in the regulation of the Wnt signalling pathway during HC formation. Moreover, WB results showed that β‐catenin protein expression was significantly upregulated in *Gprasp2*‐overexpressing organoids at D10 and D20 (Figure 4D). The protein expression of SMO and the transcription factor GLI1 was promoted by GPRASP2 during the organoid growth stage, but GLI1 protein expression was significantly reduced during the differentiation stage (Figure 4D). The knockdown of *Gprasp2* in cochlear organoids showed consistent results (Figure 4E). Notably, SMO protein levels did not decrease during the HC formation phase as GLI1 did but were consistent with β‐catenin expression (Figure 4D,E).

To further clarify the role of two transcription factors, GLI1 and β‐catenin, in the process of GPRASP2 promoting HC formation, the β‐catenin inhibitor IGC‐001 and the GLI1 inhibitor GANT61 were used with organoids during D10‐D20. To rule out effects on organoid cell survival, the maximum concentration that did not affect the proliferation of HEI‐OC1 cells after IGC‐001 administration was determined (Figure 4F). IGC‐001(1 μM) and GANT61(10 μM) were used to affect the organoid differentiation stage, and only IGC‐001 significantly reduced the number of SOX2^+^MYO7A^+^ cells (Figure 4G,H). This result suggests that the promotion of HC formation by GPRASP2 is dependent on β‐catenin but not on GLI1. Furthermore, it is clear that β‐catenin promotes cochlear SC proliferation [35], but whether the GPRASP2 promotion of cell proliferation is dependent on β‐catenin is unknown. The Count Kit‐8 (CCK‐8) assay results showed that exogenous *Ctnnb1* (β‐catenin) partially rescued cell proliferation in *Gprasp2*‐KO HEI‐OC1 cells, but not to the same extent as GPRASP2 and GLI1 (Figure S4H,I). These results suggested that GPRASP2 promoted cell proliferation mainly through GLI1, whereas the promotion of HC formation was dependent on β‐catenin.

### 3.5 GPRASP2 Regulates GLI1 and β‐Catenin Expression via SMO

**FIGURE 5:**
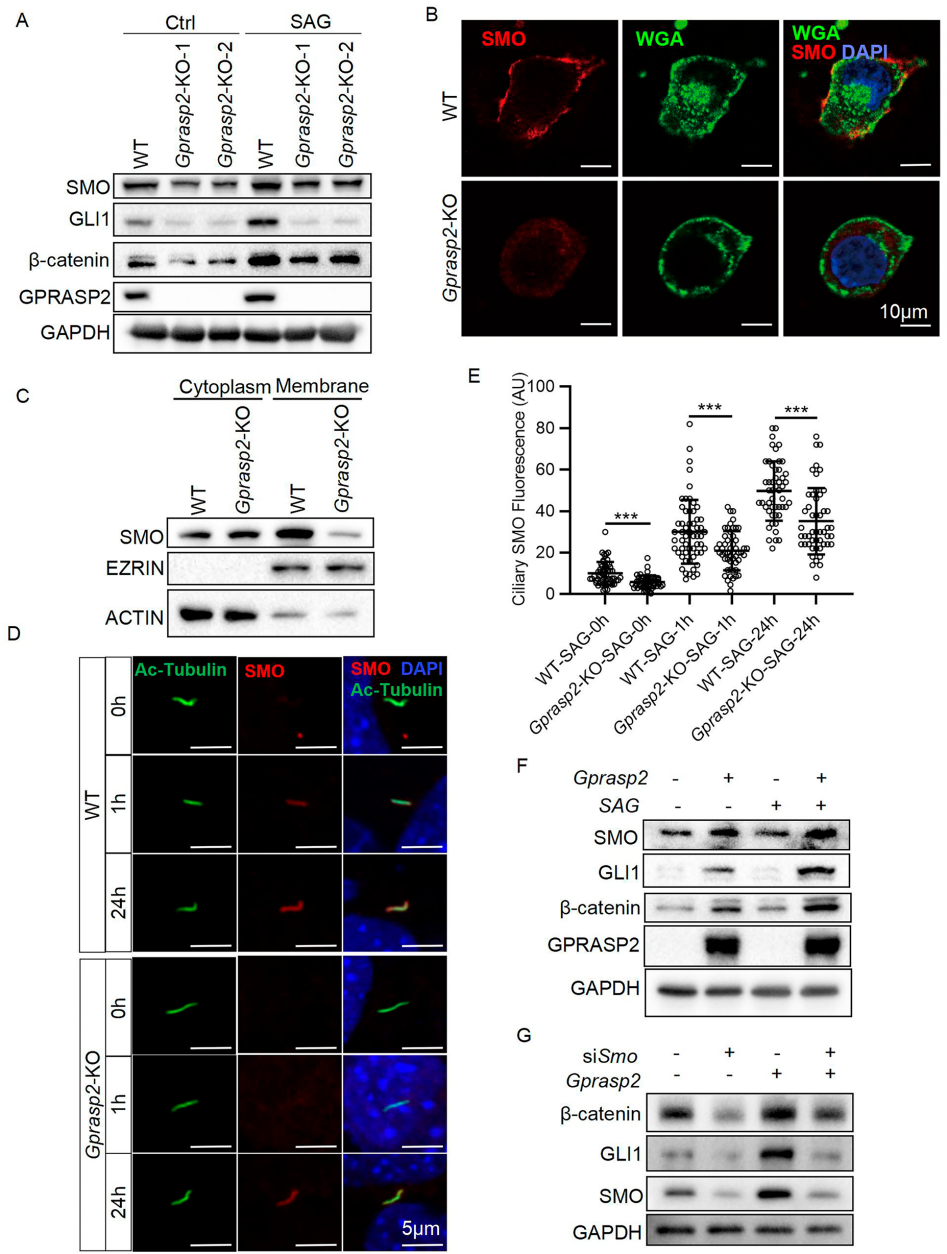
GPRASP2 regulates GLI1 and β‐catenin expression via SMO. (A) WB detection of GPRASP2, GLI1, SMO and β‐catenin protein levels in WT and *Gprasp2*‐KO HEI‐OC1 cells treated with SAG (200 nM) for 24 h. (B) Immunofluorescence staining of HEI‐OC1 cells with SMO (red), WGA (green), and DAPI (blue) without penetrating the cell membrane. Scale bar = 10 μm. (C) WB detection of SMO, EZRIN and ACTIN protein levels in the cell membrane and cytoplasm isolated from WT and *Gprasp2*‐KO HEI‐OC1 cells. (D) Immunofluorescence staining of SMO (red) and Ac‐Tubulin (green, primary cilia marker) after 1 and 24 h of SAG treatment. Scale bar = 5 μm. (E) Quantification of SMO intensity in primary cilia in (D). *n* ≥ 50, data are presented as mean ± SEM, ***p < 0.001. (F) WB detection of SMO, GLI1 and β‐catenin protein levels in *Gprasp2*‐KO HEI‐OC1 cells treated with SAG (200 nM) and *Gprasp2* plasmid alone or together. (G) WB detection of SMO, GLI1 and β‐catenin protein levels in WT HEI‐OC1 treated with si*Smo* and *Gprasp2* alone or together.

Although SMO has been reported to be a promoter of β‐catenin expression [8, 16], whether this effect is present in cochlear cells is unknown. Given the consistent expression of SMO and β‐catenin in cochlear organoids (Figure 4D,E), we further investigated whether β‐catenin is regulated by GPRASP2/SMO signalling. First, WB showed that SMO protein expression was reduced in *Gprasp2*‐KO HEI‐OC1 cells, and GLI1 and β‐catenin protein levels were significantly lower than those in WT HEI‐OC1 cells after SAG stimulation (Figure 5A). The localization of SMO proteins in the cell membrane and cilia is critical for signalling pathway responses. In our study, staining of the non‐permeabilized membrane for SMO and WGA (labelling cell membranes) in HEI‐OC1 cells showed a significant reduction in the membrane localization of SMO proteins in *Gprasp2*‐KO HEI‐OC1 cells (Figure 5B). WB analysis revealed no significant differences in cytoplasmic SMO protein levels. In contrast, the *Gprasp2*‐KO HEI‐OC1 cell membrane SMO protein level was significantly reduced compared to that in WT HEI‐OC1 cells (Figure 5C). After SAG induction, localization of SMO protein in the primary cilium of *Gprasp2*‐KO HEI‐OC1 cells significantly decreased compared to that in WT HEI‐OC1 cells, and entry into the cilia was noticeably slower (Figure 5D,E). This indicated that GPRASP2 deficiency led to a reduction in the amount of SMO protein and a reduction in its localization to the membrane. Subsequently, exogenous *Gprasp2* expression in *Gprasp2*‐KO HEI‐OC1 cells increased SMO, GLI1 and β‐catenin protein levels, with further enhancement observed upon SAG stimulation (Figure 5F), and the mRNA expression of *Lgr6* was consistent with this trend (Figure S5A). Furthermore, si*Smo* treatment of HEI‐OC1 cells led to the reduced expression of GLI1 and β‐catenin; although *Gprasp2* overexpression enhanced their expression, this effect was counteracted by si*Smo* treatment (Figure 5G), which was consistent with the mRNA expression of *Lgr6* (Figure S5B). These results suggest that GPRASP2 upregulates SMO protein level, thereby facilitating the expression of GLI1 and β‐catenin.

### 3.6 GPRASP2 Deletion Leads to Increased Lysosomal Degradation of SMO

**FIGURE 6:**
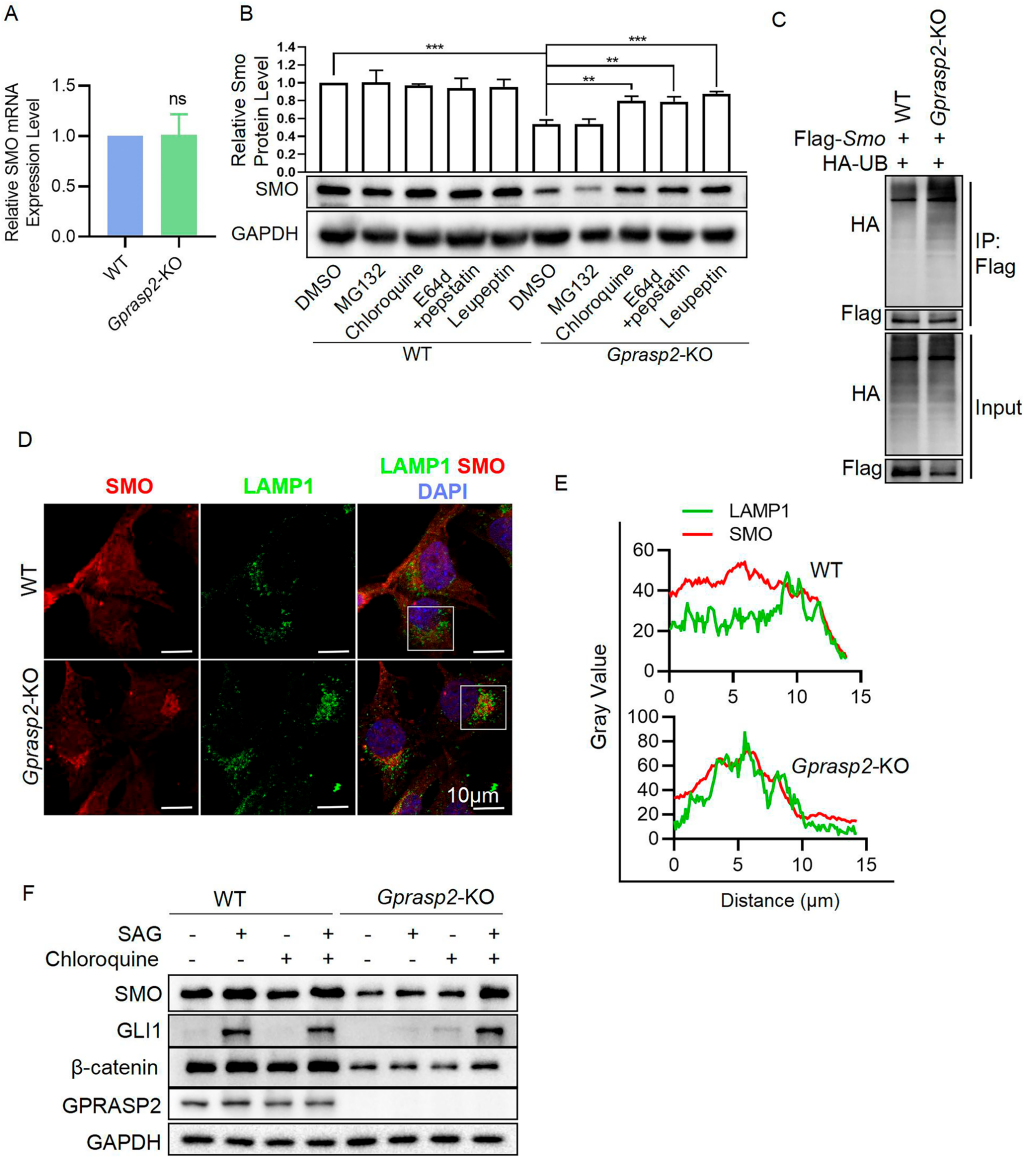
GPRASP2 deficiency leads to increased lysosomal degradation of SMO. (A) RT‐PCR detection of *Smo* mRNA in HEI‐OC1 cells. *n* = 3, data are presented as mean ± SEM. ns, not significant. (B) WB detection of SMO protein levels in WT and *Gprasp2*‐KO HEI‐OC1 cells treated with chloroquine (100 μM), pepstatin (100 nM)‐E64d (5 μM), leupeptin (10 μM) or MG132 (5 μM) for 12 h. (C) Ubiquitination assays of SMO protein were performed by immunoprecipitation and WB in WT and *Gprasp2*‐KO HEI‐OC1 cells transfected with the HA‐UB and Flag‐*Smo* plasmid. (D) Immunofluorescence staining of HEI‐OC1 cells with SMO (red), LAMP1 (green) and DAPI (blue) treated with chloroquine for 12 h. Scale bar = 10 μm. (E) Fluorescence value distribution of LAMPK and SMO within the white box in (D). (F) WB detection of SMO, GLI1, β‐catenin and GPRASP2 protein levels in WT and *Gprasp2*‐KO HEI‐OC1 cells treated with chloroquine and SAG alone or together.

The above results show that GPRASP2 regulates SMO expression, but the underlying molecular mechanism is unknown. First, to determine whether GPRASP2 regulates SMO protein expression at the transcriptional or post‐transcriptional level, *Smo* mRNA levels were compared in WT and *Gprasp2*‐KO HEI‐OC1 cells (Figure 6A). This suggests that regulation of the SMO protein by GPRASP2 occurred at the post‐transcriptional level. Furthermore, members of the GPRASP family play crucial roles in the transport of GPCRs, which can lead to alterations in protein fate. Proteins that cannot be sorted or transported correctly are frequently degraded. Therefore, the lysosomal inhibitors (chloroquine, pepstatin/E64‐d and leupeptin) and the proteasomal inhibitor (MG132) were used to block protein degradation in both WT and *Gprasp2*‐KO HEI‐OC1 cells. The effective concentration of MG132 was determined by detecting ubiquitinated (UB) proteins, as its inhibition of the proteasome leads to their accumulation. The effective concentrations of chloroquine, pepstatin/E64‐d, and leupeptin were determined by measuring the autophagy‐related protein sequestosome 1(SQSTM1/P62), whose accumulation results from blocked lysosomal degradation (Figure S6). WB assay showed that GPRASP2 deficiency increased SMO degradation via the lysosomal pathway (Figure 6B). According to previous reports, SMO protein is degraded after ubiquitination labelling [36, 37], and we found that ubiquitinated SMO levels also increased in KO cells by Co‐IP analysis (Figure 6C). Consistently, following chloroquine treatment to inhibit SMO degradation, IF results revealed a distinct aggregation of SMO near the nuclear periphery of *Gprasp2*‐KO HEI‐OC1 cells, closely corresponding to the location marked by LAMP1 (Figure 6D,E). The treatment of the SMO agonist (SAG) in *Gprasp2*‐KO HEI‐OC1 cells did not upregulate GLI1 and β‐catenin protein levels, but these two proteins were significantly increased after the addition of chloroquine to block SMO protein degradation (Figure 6F). These results indicate that GPRASP2 deficiency leads to increased lysosomal degradation of SMO protein, which affects the protein expression of GLI1 and β‐catenin.

### 3.7 SAG Further Enhances GPRASP2 to Promote HC Formation in Cochlear Explants

**FIGURE 7:**
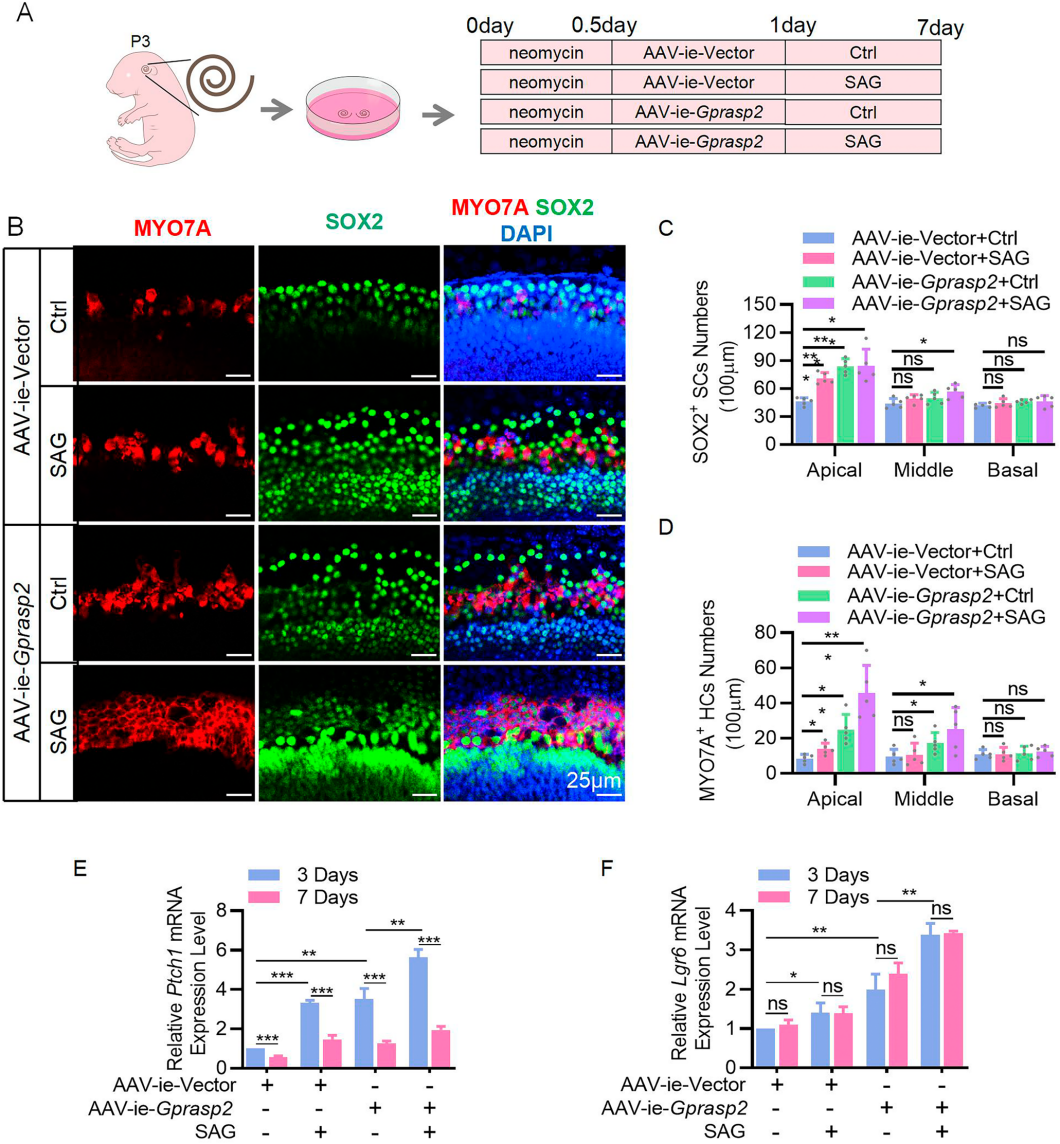
SAG further enhances GPRASP2 to promote HC formation in cochlear explants. (A) Experimental scheme of cochlear explants transfected AAV‐ie‐*Gprasp2* and treated with neomycin (0.5 mM) and SAG (200 nM). (B) Immunofluorescence staining of apical‐turn cochlear explants with MYO7A (red), SOX2 (green) and DAPI (blue). Scale bar = 25 μm. (C, D) Quantification of MYO7A^+^ cells and SOX2^+^ cells within the cochlear sensory domain in (B). *n* ≥ 5. RT‐PCR detection of *Ptch1* (E) and *Lgr6* (F) mRNAs in cochlear explants transfected with AAV‐ie‐Vector or AAV‐ie‐*Gprasp2*. *n* = 3, each sample was generated by 16 cochlear explants.

The above results suggest that SMO may have an important role in GPRASP2's promotion of SC proliferation and HC formation, coupled with reports that SMO activation significantly promotes the proliferation of progenitor cells in cochlear explants damaged by neomycin [15]. Therefore, we considered whether GPRASP2 would have the same effect and whether the SMO agonist SAG could further strengthen this effect. In subsequent experiments, after 12 h of neomycin injury, cochlear explants were transfected with AAV‐ie‐*Gprasp2* and supplemented with SAG (Figure 7A). The results demonstrated that the number of SCs and HCs in the apical turn of the cochlear explants in the AAV‐ie‐*Gprasp2* group increased. SAG treatment further enhanced this effect; however, this effect was not observed in the basal turn of cochlear explants (Figure 7B–D). In addition, *Gprasp2* overexpression combined with SAG treatment could synergically promote the expression of Ptch1 and Lgr6 on Day 3. Notably, the expression of *Ptch1* on Day 7 was significantly lower than that on Day 3, whereas the expression of *Lgr6* showed no significant difference (Figure 7E,F). These results suggested that the role of GPRASP2 during HC formation was more dependent on β‐catenin.

## 4 Discussion

Our study showed that GPRASP2 plays a crucial role in the proliferation of cochlear SCs and subsequently contributes to HC formation. Mature HCs within the mammalian cochlea exhibit a limited capacity for spontaneous regeneration following HC damage or loss induced by noise, drugs or ageing [38, 39, 40]. Given that both SCs and HCs originate from common progenitor cells, the activation of some genes prompts SCs to re‐enter the cell cycle and re‐differentiate into HCs, making SCs a potential target for HC regeneration [41, 42]. This establishes a theoretical foundation for considering *GPRASP2* as a potential target for hearing loss caused by HC deficiency.

This study revealed that GPRASP2 regulates the Hedgehog signalling pathway to promote the proliferation of SCs and the Hedgehog pathway is essential for the proliferation of cochlear progenitor cells. Hedgehog is not only critical for cochlear tube lengthening and for controlling the timing of HC differentiation in the development [11, 28], but also this pathway activation maintains the SOX2‐expressing prosensory cell progenitor state [27]. This provides a theoretical basis for the involvement of GPRASP2 in SOX2^+^ cell proliferation mediated by SMO‐GLI1 signalling. Notably, an increase in SMO expression simultaneously promotes the proliferation of progenitor cells and subsequent HC formation in cochlear implants following neomycin‐induced damage [29], which seems to contradict the sustained expression of Shh inhibiting HC differentiation. However, a *Drosophila* model showed that intracellular SMO is activated independently of Shh stimulation and initiates downstream gene expression [43]. SAG activation of SMO can also activate other effector molecules in addition to GLI1 [44]. This may be the reason why Shh activates the Hedgehog signalling pathway and SMO overexpression has different effects on HC differentiation.

Our results indicated that the different roles of SMO in SC proliferation and HC formation could be due to the ability of SMO to modulate other effector molecules. Although the classical Hedgehog signalling pathway is dependent on GLI1, it has been reported that the suppression of SMO expression reduces β‐catenin protein levels and nuclear localization, inhibiting intestinal tumorigenesis [45]. In our study, GPRASP2‐mediated SMO/β‐catenin signalling regulated HC formation, but other effector molecules involved in this process are not yet clear. Notably, this study showed that GPRASP2 promoted the expression of the β‐catenin‐target gene, *Lgr6*; this process depended on SMO in HEI‐OC1. LGR6^+^ cells represent a subpopulation of LGR5^+^ progenitors, which have a greater proliferative capacity than LGR6^+^ progenitors, whereas LGR6^+^ progenitors are an enriched population of HC progenitor cells [46]. However, whether GPRASP2 promotes HC formation depends on the expression of LGR6 needs further study.

We have shown that GPRASP2 deficiency leads to SMO protein degradation but results in abnormal aggregation in the cytoplasm. In addition, GPRASP2 deficiency resulted in a decrease in the amount of SMO protein in the cell membrane, but there was no significant change in the cytoplasm. This may be due to the fact that GPRASP2 is involved in the sorting and trafficking of SMO proteins before SMO localization to the cell membrane. In addition, we found that even after blocking the degradation of SMO, the levels of GLI1 and β‐catenin proteins did not increase. This further suggested that GPRASP2 might have been involved in the transport or sorting process of the SMO. Specifically, the release of SMO protein from the Golgi and endoplasmic reticulum requires dissociation from transmembrane p24 trafficking protein 2 (TMED2) protein [47]. If the SMO‐TMED2 complex cannot be dissociated, the SMO protein is restricted to the Golgi and endoplasmic reticulum and eventually degraded by lysosomes. As speculated, partial colocalization of the abnormally aggregated SMO protein with TMED2 was observed in *Gprasp2*‐KO HEI‐OC1 cells via IF analysis (Figure S7A). Co‐IP was performed to determine whether GPRASP2 regulates the dissociation of SMO and TMED2. Significant dissociation of the SMO‐TMED2 complex was observed upon Shh stimulation in WT HEI‐OC1 cells, which is consistent with previous findings. Conversely, GPRASP2 deficiency inhibited the release of SMO protein from the SMO‐TMED2 complex (Figure S7B). In the inactive state of Hedgehog, the binding between SMO and TMED2 in *Gprasp2*‐KO HEI‐OC1 cells was significantly increased compared to that in WT HEI‐OC1 cells, and this effect was rescued by *Gprasp2* supplementation (Figure S7B,C). Collectively, these findings indicate that GPRASP2 promoted the dissociation of SMO‐TMED2. Without GPRASP2, SMO and TMED2 dissociation is impeded, leading to subsequent lysosomal degradation. Consistent with this, Tmed2 acts upstream of both the GLI‐dependent and GLI‐independent effects of SMO [47].

Our study showed that GPRASP2/SMO signalling regulates different effector molecules at different stages, promoting SC proliferation mainly through GLI1, whereas it promoted β‐catenin effects during HC formation. Therefore, *GPRASP2* is a promising target for HC regeneration. Currently, AAV vectors via cochlear injections or cerebrospinal fluid delivery are primarily utilised in the study of gene therapy for genetic hearing loss [20, 48, 49, 50]. Significant improvements in hearing were detected in two deaf patients with *OTOF* mutations following AAV‐OTOF treatment [49, 51]. Therefore, it is important to find more gene therapy candidates for the treatment of deafness. Our study suggests that the delivery of AAV‐*GPRASP2* in cochleae might be a promising approach for the treatment of genetic hearing loss.

## 5 Conclusions

**FIGURE 8:**
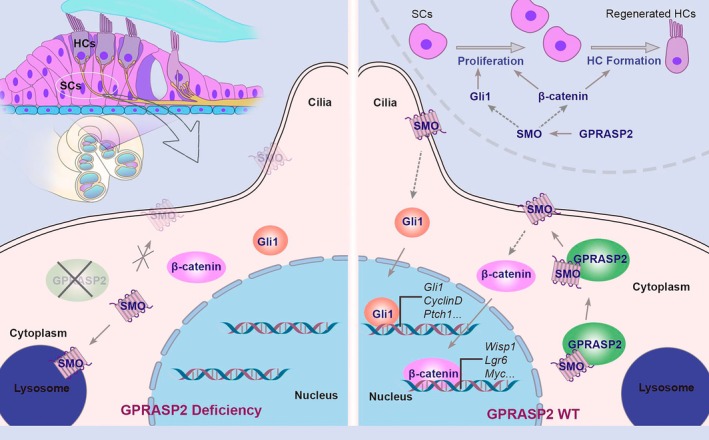
Schematic diagram of GPRASP2‐mediated SC proliferation and HC formation. GPRASP2 deficiency results in increased lysosomal degradation of SMO. GPRASP2‐mediated SMO/GLI1 signalling promotes SC proliferation, which contributes to HC formation. GPRASP2‐mediated SMO/β‐catenin signalling is implicated in HC fate specification and differentiation.

In summary, this study revealed that GPRASP2 upregulated SMO to promote the expression of the transcription factors GLI1 and β‐catenin, promoting SMO/GLI1‐mediated SC proliferation and SMO/β‐catenin‐mediated HC formation (Figure 8). These findings indicate that GPRASP2 could be a promising target for HC regeneration and in vivo delivery of AAV‐*Gprasp2* to the cochlea could be a potential strategy for HC regeneration and auditory rehabilitation, which would be further investigated in our future work.

## Author Contributions

Q.W., J.Y., and X.C. participated in the study conception and design. J.C., K.H. and W.L. performed the experiments and/or collected data. equally contributed to investigation, data analysis, and original draft preparation. T.W., S.Y., Z.C., G.X. and Q.W. contributed to data analysis and interpretation. J.C. drafted the manuscript. J.Y. and X.C. critically revised and edit the manuscript. All authors read and approved the final manuscript.

## Ethics Statement

All animal studies were reviewed and approved by the Institutional Animal Care and Use Committee of Nanjing Medical University.

## Conflicts of Interest

The authors declare no conflicts of interest.

## Supporting information


**Data S1.** Supporting Information.

## Data Availability

All data generated or analysed during this study are included in this published article and its Supporting Information files. The data that support the findings of this study are available from the corresponding author upon reasonable request.
