# Supplementary material for: Implication of GPRASP2 in the Proliferation and Hair Cell‐Forming of Cochlear Supporting Cells
Source: Cell Prolif. 2024 Dec 15;58(11):e13792. doi: 10.1111/cpr.13792 (PMC12584871; doi:10.1111/cpr.13792)
Supplement: Supplementary file 1 — Data S1. Supporting Information. [file CPR-58-e13792-s001.docx]

**Implication of GPRASP2 in the proliferation and hair cell-forming of cochlear supporting cells**

**Jing Cai^1^, Kun Huang^1^, Wenrui Li^1^, Tianming Wang^2^, Shen Yue^1^, Zhibin Chen^4^, Guangqian Xing^4^, Qinjun Wei^1, 3*^, Jun Yao^1, 3*^, Xin Cao^1, 3*^**

^1^Department of Medical Genetics, School of Basic Medical Science, Nanjing Medical University, Nanjing, China

^2^Central Laboratory, Translational Medicine Research Center, the affiliated Jiangning Hospital of Nanjing Medical University, Nanjing, China

^3^Jiangsu Key Laboratory of Xenotransplantation, Nanjing Medical University, Nanjing, China

^4^Department of Otolaryngology, the First Affiliated Hospital with Nanjing Medical University, Nanjing, China

^*^**Corresponding author**: Qinjun Wei

Department of Medical Genetics, School of Basic Medical Science, Nanjing Medical University

No.101 Longmian Avenue, Nanjing, China 211166

Tel/Fax: +86-25-86869413

E-mail address: wqj@njmu.edu.cn (Q. Wei)

^*^**Corresponding author**: Jun Yao

Department of Medical Genetics, School of Basic Medical Science, Nanjing Medical University

No.101 Longmian Avenue, Nanjing, China 211166

Tel/Fax: +86-25-86869413

E-mail address: joelyao@njmu.edu.cn (J. Yao)

^*^**Corresponding author**: Xin Cao

Department of Medical Genetics, School of Basic Medical Science, Nanjing Medical University

No.101 Longmian Avenue, Nanjing, China 211166

Tel/Fax: +86-25-86869341

E-mail address: caoxin@njmu.edu.cn (X. Cao)

**Running title:** *GPRASP2 promotes SCs proliferation and HCs formation*

**Supplementary Table:**

| Table S1 Primer and siRNA sequences | | |
| --- | --- | --- |
| Gene | Forward 5’→ 3’ | Reverse 5’→ 3’ |
| *Gprasp2* | GAATCGAGCCCTGGTTTGGA | TTTGGATCTGGGCTTCGACC |
| *Gli1* | CCAAGCCAACTTTATGTCAGGG | AGCCCGCTTCTTTGTTAATTTGA |
| *Ptch1* | AAAGAACTGCGGCAAGTTTTTG | CTTCTCCTATCTTCTGACGGGT |
| *Sox2* | ATGACCAGCTCGCAGACCTAC | GCCTCGGACTTGACCACAGAG |
| *Myo7A* | GGATGTGCGAGATGCCTTTG | GCTTGTAGATTGCTGCGTTGA |
| *Atoh1* | TGGTAAGGAGAAGCGGCTGTG | GCCAAGCTCGTCCACTACAAC |
| *Pou4f3* | TCTGGCGGCGGTGGATAT | GCTGCTCATGGTATGGTAGGT |
| *Lgr6* | CATAACAACCGCATCCAGCA | CTGCCCATGAAGGCTTTCTC |
| *Wisp1* | CACTCGGATCTCTAACGTCAATGC | CCTGCCTTGATGTGTAGTTGGATG |
| *Nrarp* | TGGTGAAGCTGTTGGTCAAGTTC | GATGAGATAGAGCACGATGTCCTG |
| *Hes1* | GCCAATTTGCCTTTCTCATCCC | CTGGAAGGTGACACTGCGTTAG |
| *Trim71* | GAAGTGAGCGACCAGCAGAATG | CGACTTGACCACGACCTTGAAG |
| *Hmga2* | CCCAGAAGAAAGCAGAGACCATTG | GGGCAGACTTGAGGTTTGTGATTC |
| siRNA | Sense 5’→ 3’ | Antisense 5’→ 3’ |
| Si*Smo* | GUGGGAUUCAGUGUCAGAATT | UUCUGACACUGAAUCCCACTT |

| Table S2. Details of reagents used in the study | | | |
| --- | --- | --- | --- |
| **Reagent Name** | **Vendor** | **Cat#** | **Final Concentration** |
| Penicillin G | MCE | HY-N7139 | 50 IU/mL |
| GDC-0449 | MCE | HY-10440 | 6 μM; 1 μM |
| SAG | MCE | HY-10440 | 200nM |
| IGC-001 | MCE | HY-14428 | 1 μM |
| B27 Supplement | Gibco | 17504044 | 1:50 |
| N2 supplement | WISENT | 305-016-IL | 1:100 |
| Glutamax I | Gibco | A1286001 | 1:100 |
| Laminin | absin | abs45129192 | 0.02mg/ml |
| Murine EGF | absin | abs01015 | 50 ng/ml |
| Murine FGF | MCE | HY-P7065 | 50 ng/ml |
| Murine IGF-1 | MCE | HY-P7070 | 50 ng/ml |
| CHIR99021 | MCE | HY-0182 | 3 µM |
| 616452 | MCE | HY-13012 | 2 µM |
| L-ascorbic acid 2-phosphate (pVc) | MCE | HY-103701 | 100 µg/ml |
| Valproic Acid Sodium Salt（VPA） | MCE | HY-0585 | 1 mM |
| LY411575 | MCE | HY-50752 | 10 µM |
| DNAse I | sigma | D4513 | 200 units/ml |
| Matrigel | absin | Abs9490 | 2% |
| Trypsin inhibitor | Diamond | A003587-0001 | 20 mg/ml |
| Neomycin | MCE | HY-B0470 | 0.5 mM |
| Collagen Type 1 | Corning | 354236 | - |
| Organoid Digestion Solution | absin | abs9520 | - |
| HBSS | Servicebio | G4203 | - |
| DMEM | WISENT | 319-005-CL | - |
| DMEM/F12 | Gibco | C11330500BT | - |
| 0.25% trypsin-EDTA | gibco | 25200072 | - |
| Fetal Bovine Serum（FBS） | ExCell | fsp500 | - |

**Supplementary Figures:**


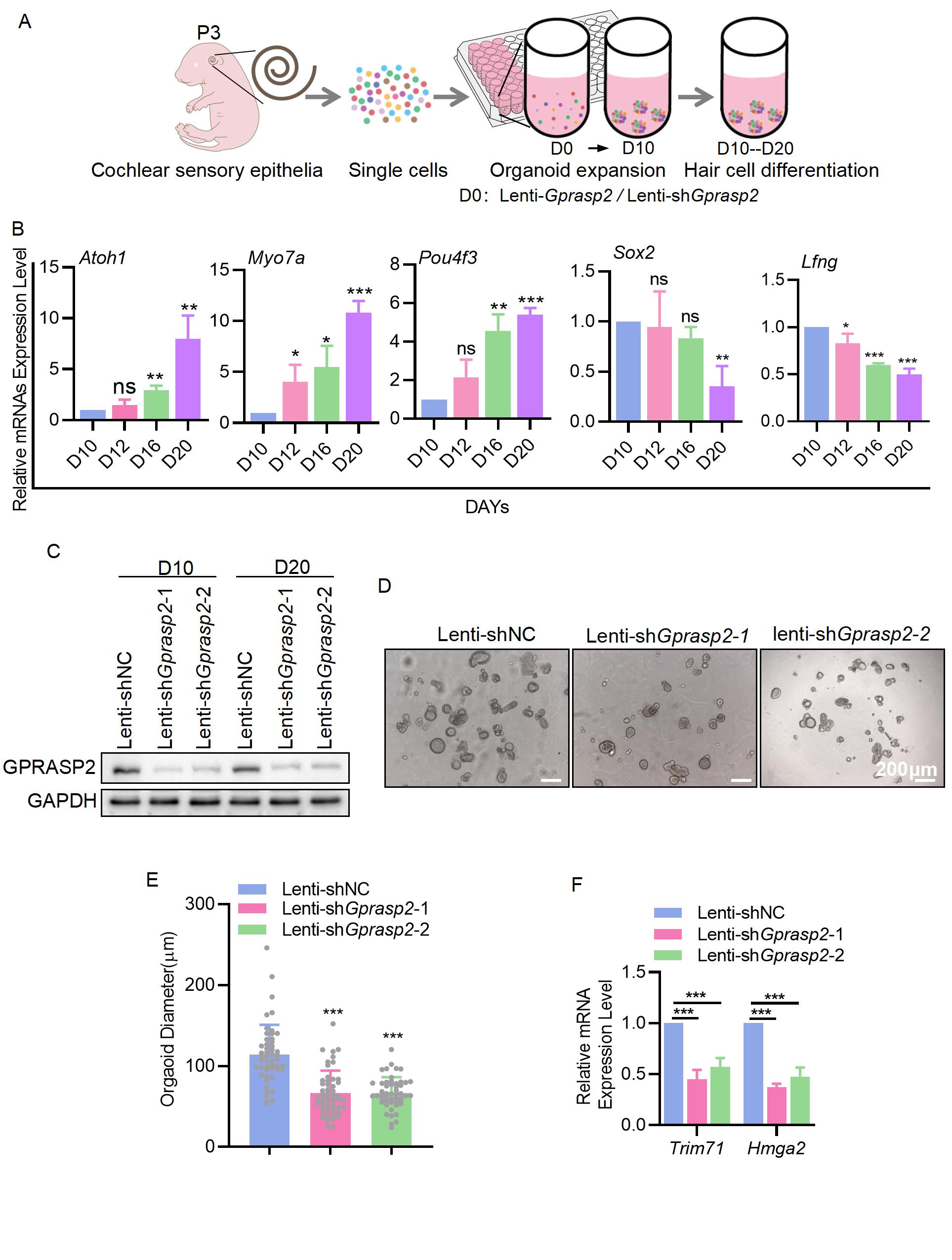


**Figure S1. Knockdown of *Gprasp2* inhibits cochlear organoid growth.** (A) Experimental scheme of cochlear organoid establishment, expansion, and differentiation. D, day. (B) RT-PCR detection of mRNA levels of HCs genes (*Atoh1, Pou4f3,* and *Myo7a*) and SCs genes (*Sox2* and *Lfng*) in organoids at D10, D12, D16, and D20. n=3, each sample contains roughly 150 organoids. (C) WB detection of GPRASP2 and GAPDH protein levels in cochlear organoids transfected with Lenti-shNC, Lenti-sh*Gprasp2*-1, or Lenti-sh*Gprasp2*-2. (D) Representative images of cochlear organoids transfected with Lenti-shNC, Lenti-sh*Gprasp2*-1 or Lenti-sh*Gprasp2*-2. Scale bars, 200 μm. (E) Diameter of organoids in panel (D). n≥50. (F) RT-PCR detection of *Trim71* and *Hmga2* mRNAs in cochlear organoids transfected with Lenti-shNC, Lenti-sh*Gprasp2*-1, or Lenti-sh*Gprasp2*-2. n=3, each sample contains roughly 150 organoids. Data in bar graphs are presented as mean ± SEM. ** P < 0.01, *** P < 0.001.


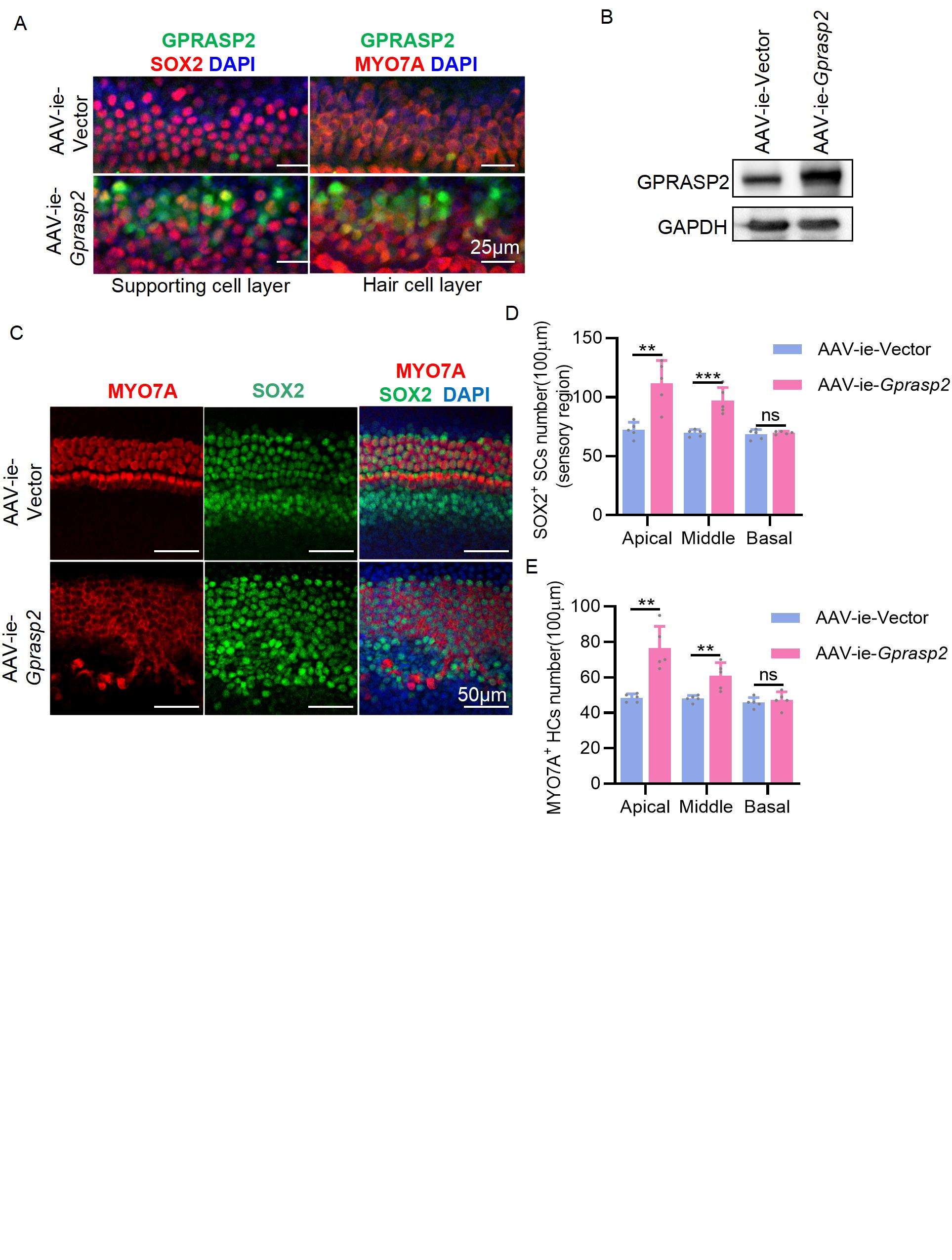


**Figure S2. AAV-ie-*Gprasp2* promotes HCs formation in cochlear explants.** (A) and (B) Efficiency of AAV-ie-*Gprasp2* infection of cochlear explants was validated by immunofluorescence staining with GPRASP2(green), SOX2(red), MYO7A(red) and DAPI (blue) and WB analysis with GPRASP2 and GAPDH. Scale bars, 25 μm. (C) Immunofluorescence staining of apical-turn cochlear explants with MYO7A (red), SOX2 (green), and DAPI (blue) after transduction with AAV-ie-Vector or AAV-ie-*Gprasp2* for 6 days. Scale bars, 50 μm. (D) and(E) Quantification of bright MYO7A+ cells and SOX2^+^ cells within the cochlear sensory domain in panel (C). n≥5. Data in bar graphs are presented as mean ± SEM. ** P < 0.01, *** P < 0.001, ns. not significant.


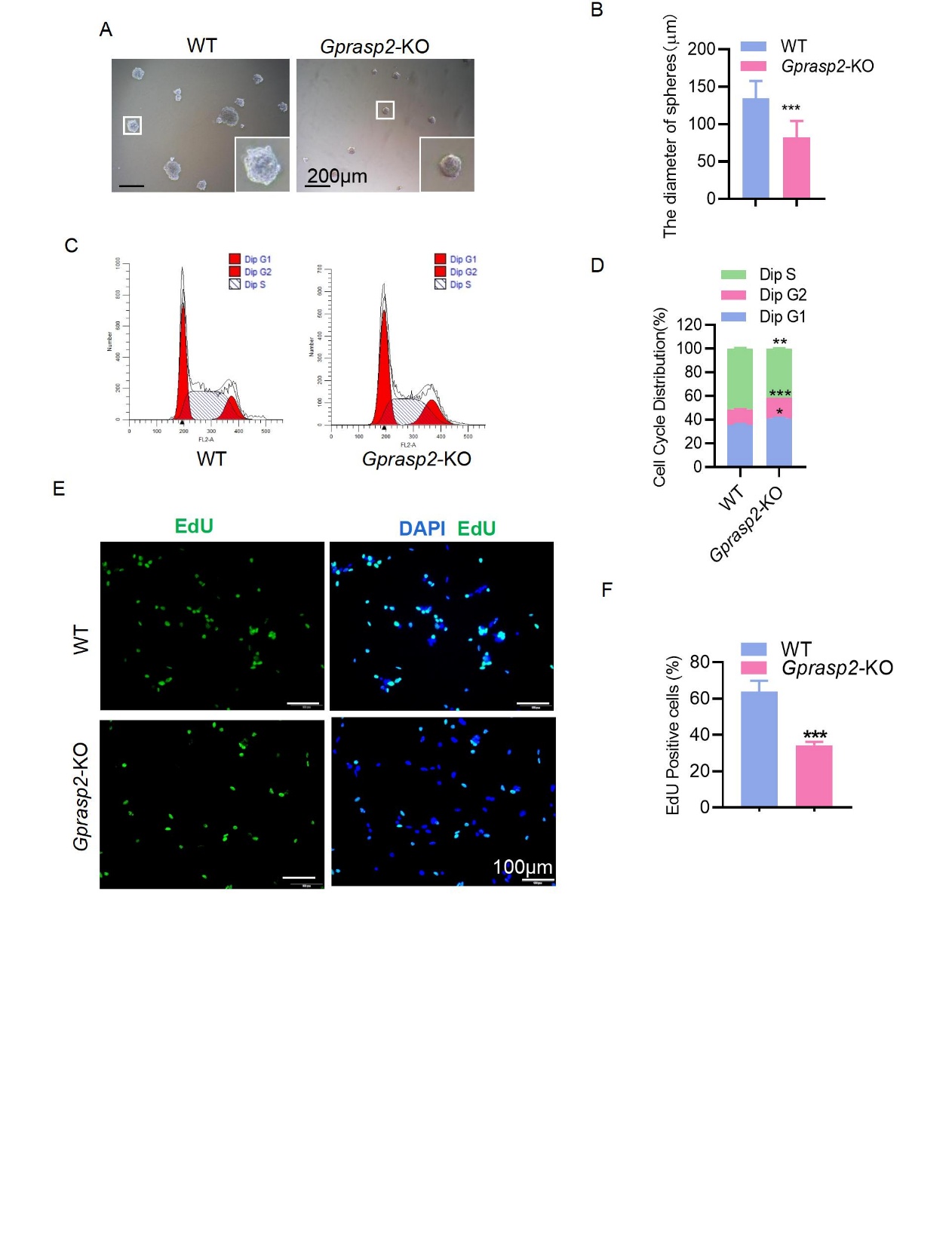


**Figure S3. The proliferation ability of *Gprasp2*-KO HEI-OC1 cells decreased.** (A)Representative images of spheres formed by WT and *Gprasp2*-KO HEI-OC1 cells. Scale bars, 200 μm. (B) Diameter of spheres in panel (A). n≥50. (C)The cell cycle of WT and *Gprasp2*-KO HEI-OC1 cells was detected by flow cytometry. (D) Quantification of three independent experiments as in panel (C). (E) Immunofluorescence staining of WT and *Gprasp2*-KO HEI-OC1 cells with EdU (green) and DAPI (blue). Scale bars, 100 μm. (F) Percentage quantification of EdU^+^ cells per field in panel (E). n=4.


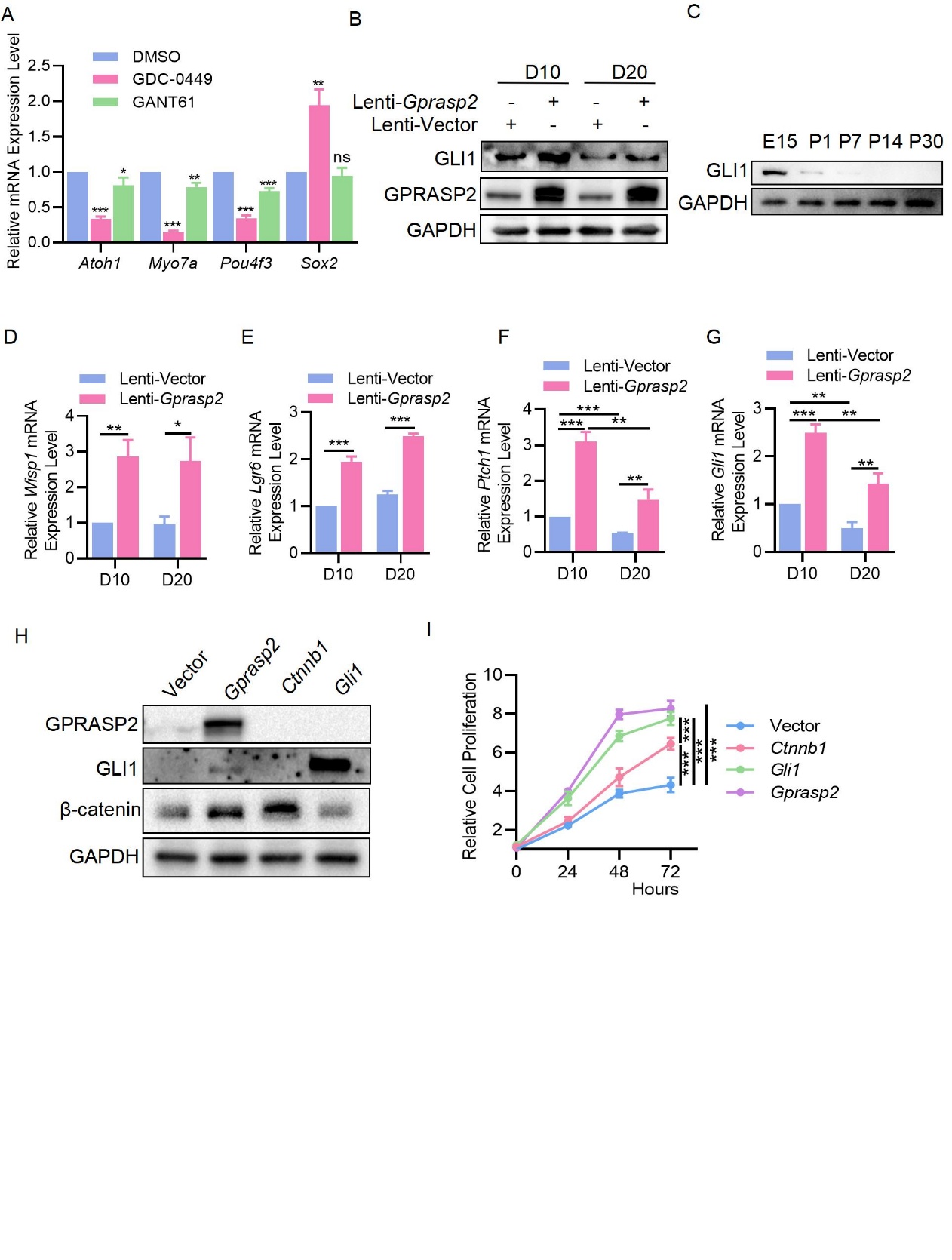


**Figure S4. GPRASP2 promotes cell proliferation mainly depends on GLI1, but GLI1 expression decreases during cochlear organoid differentiation.** (A) RT-PCR detection of *Atoh1*, *Myo7a*, *Pou4f3,* and *Sox2* mRNAs in cochlear organoids at D20 treated with DMSO (1:1000), GDC-0449(1μM), or GANT61(10μM) from D10. n=3, each sample was generated by approximately 150 organoids. (B) WB detection of GPRASP2 and GLI1 protein levels in cochlear organoids transfected with Lenti-vector or Lenti-*Gprasp2* at D10 and D20. Each sample was generated by approximately 150 organoids. (C) GLI1 protein levels in the mouse cochlea at E15, P1, P7, P14, and P30, and GAPDH were used as an internal control. RT-PCR detection of β-catenin target genes *Wisp1*(D), *Lgr6*(E), *Ptch1*(F) and *Gli1*(G) mRNAs in cochlear organoids transfected with Lenti-Vector or Lenti-*Gprasp2*. n=3, each sample was generated by approximately 150 organoids. (H) WB detection of GPRASP2, GLI1, and β-catenin protein levels in *Gprasp2*-KO HEI-OC1 cells transfected with *Gprasp2*, *Gli1*, or *Ctnnb1* plasmid. (I) Relative cell proliferation of *Gprasp2*-KO HEI-OC1 cells transfected with *Gprasp2*, *Gli1*, or *Ctnnb1* plasmid was assessed by CCK-8. Data in bar graphs are presented as mean ± SEM. * P < 0.05, ** P < 0.01, *** P < 0.001.


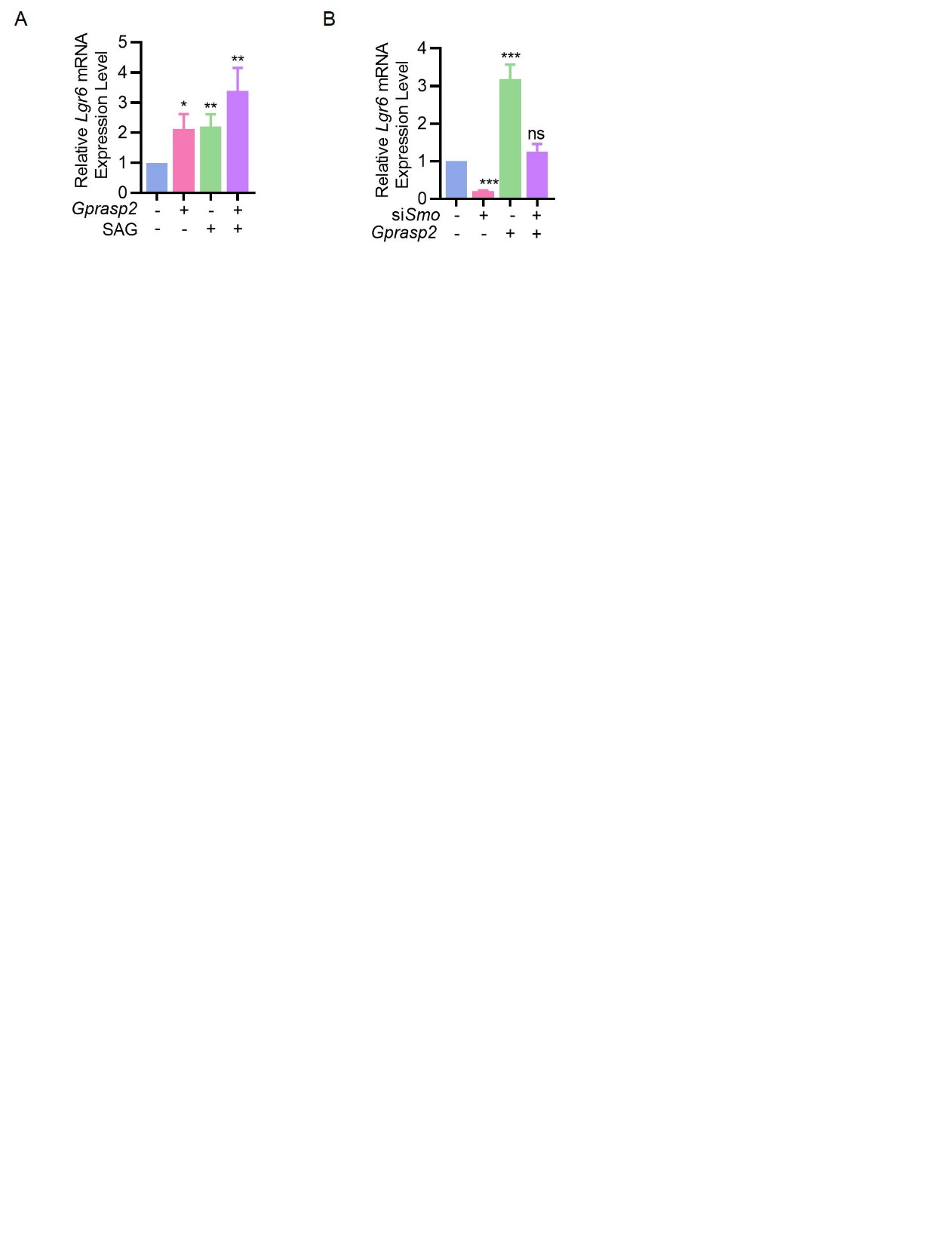


**Figure S5. The regulation of Lgr6 mRNA expression by GPRASP2 is dependent on SMO.** (A) RT-PCR detection of *Lgr6* mRNA in *Gprasp2*-KO HEI-OC1 cells treated with SAG (200 nM) and *Gprasp2* plasmid alone or together. (B) RT-PCR detection of *Lgr6* mRNA in WT HEI-OC1 treated with si*Smo* and *Gprasp2* alone or together.


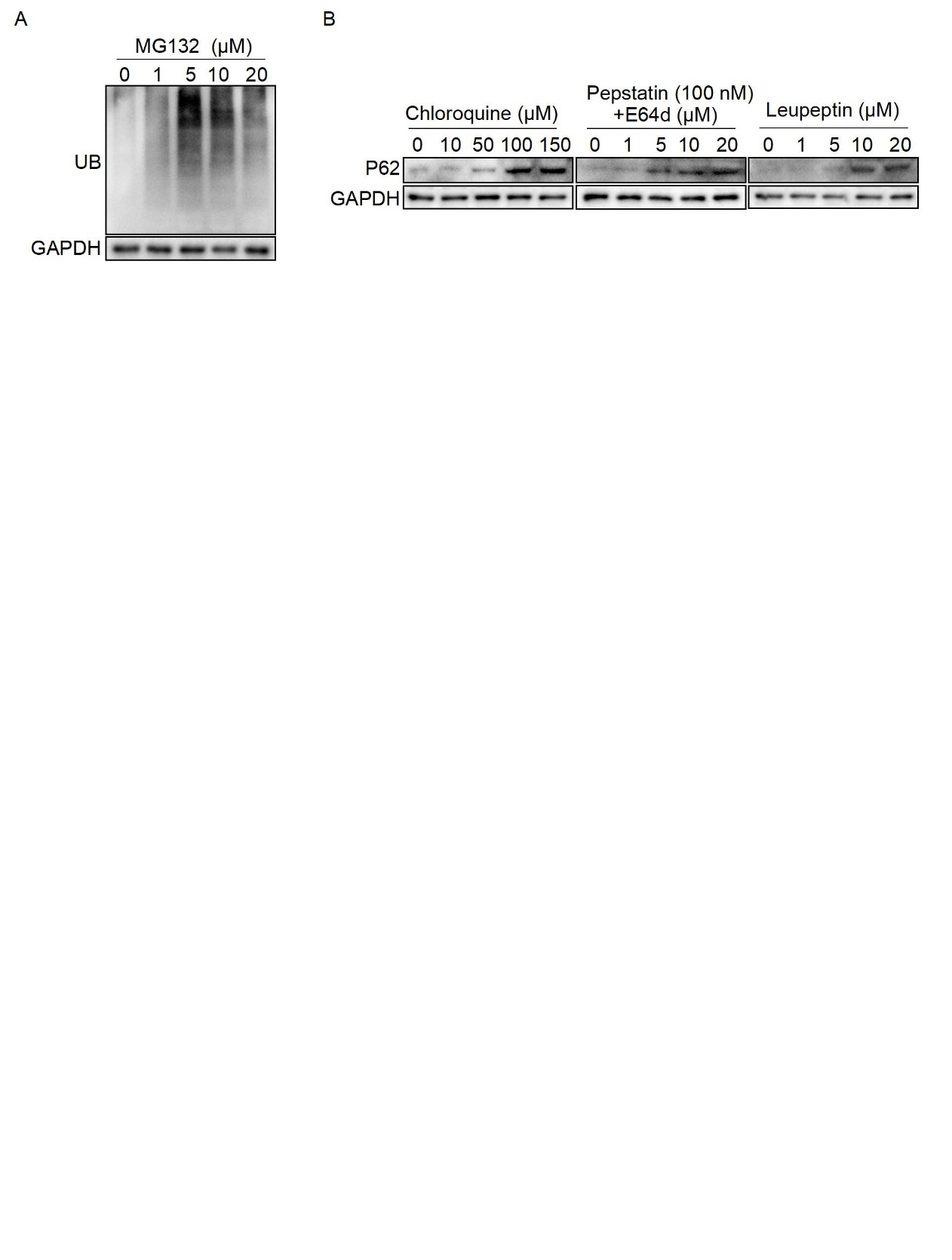
**Figure S6. Effect validation of proteasome and lysosome inhibitors.** (A) WB detection of UB in WT HEI-OC1 cells treated with different concentrations of MG132. (B) WB detection of P62 in WT HEI-OC1 cells treated with different concentrations of MG132 chloroquine, pepstatin/E64D or leupeptin.


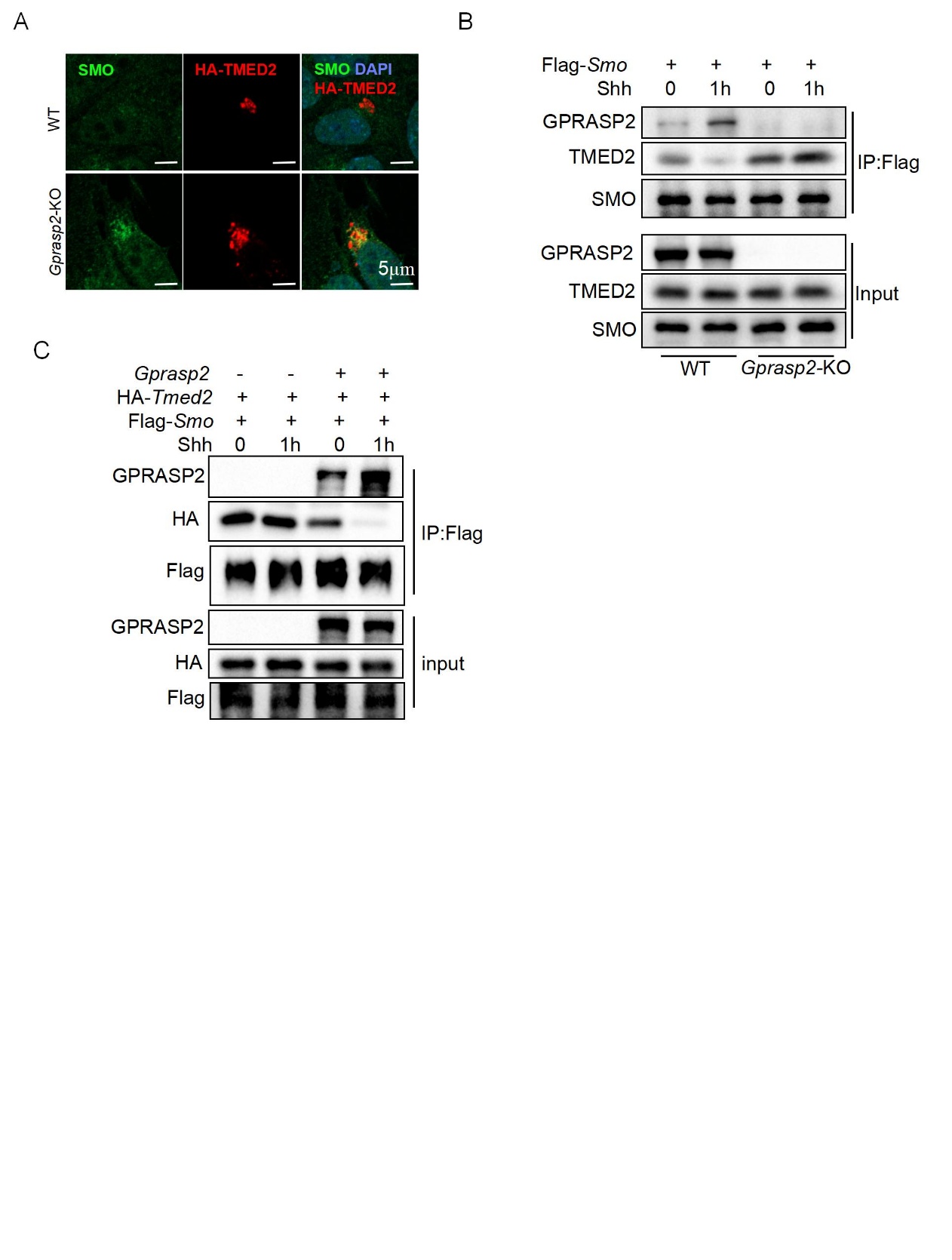


**Figure S7. GPRASP2 promotes the dissociation of SMO-TMED2 complex.** (A) Immunofluorescence staining of HEI-OC1 cells with SMO (green), HA (red), and DAPI (blue) treated with chloroquine for 12 hours. Scale bars, 5 μm. (B) Co-immunoprecipitation analyses of the SMO-GPRASP2 and SMO-TMED2 interaction in WT and *Gprasp2*-KO HEI-OC1 cells transfected with Flag-*Smo* plasmids and treated with Shh for 1 hour before immunoprecipitation. Immunocomplexes were precipitated using anti-Flag and blotted with SMO, TMED2 and GPRASP2. (C) Co-immunoprecipitation analysis of the SMO-GPRASP2 and SMO-TMED2 interaction in *Gprasp2*-KO HEI-OC1 cells transfected with HA-*Tmed* and Flag-*Smo* or supplemented with *Gprasp2* plasmids.
